# Impact of Intracellular Proteins on μ-Opioid Receptor Structure and Ligand Binding

**DOI:** 10.1021/acs.jpcb.4c05214

**Published:** 2024-12-19

**Authors:** Caitlin E. Scott, Leah A. Juechter, Josephine Rocha, Lauren D. Jones, Brenna Outten, Taylor D. Aishman, Alaina R. Ivers, George C. Shields

**Affiliations:** † Department of Chemistry and Biochemistry, California State University, Los Angeles, California, 90032, United States; ‡ Department of Chemistry, Hendrix College, Conway, Arkansas 72032, United States; § Department of Chemistry, Furman University, Greenville, South Carolina 29613, United States

## Abstract

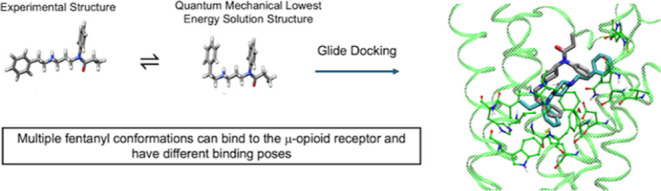

Chronic pain is a prevalent problem affecting approximately one out of every five adults in the U.S. The most effective way to treat chronic pain is with opioids, but they cause dangerous side effects such as tolerance, addiction, and respiratory depression, which makes them quite deadly. Opioids, such as fentanyl, target the μ-opioid receptor (MOR), which can then bind to the intracellular G_i_ protein or the β-arrestin protein. The G_i_ pathway is primarily responsible for pain relief and potential side effects, but the β-arrestin pathway is chiefly responsible for the unwanted side effects. Ideally, an effective pain medication without side effects would bind to MOR, which would bias signaling solely through the G_i_ pathway. We used the Bio3D library to conduct principal component analysis to compare the cryo-electron microscopy MOR structure in complex with the G_i_ versus an X-ray crystallography MOR structure with a nanobody acting as a G_i_ mimic. Our results agree with a previous study by Munro, which concluded that nanobody-bound MOR is structurally different than G_i_-bound MOR. Furthermore, we investigated the structural diversity of opioids that can bind to MOR. Quantum mechanical calculations show that the low energy solution structures of fentanyl differ from the one bound to MOR in the experimental structure, and p*K*_a_ calculations reveal that fentanyl is protonated in aqueous solution. Glide docking studies show that higher energy structures of fentanyl in solution form favorable docking complexes with MOR. Our calculations show the relative abundance of each fentanyl conformation in solution as well as the energetic barriers that need to be overcome to bind to MOR. Docking studies confirm that multiple fentanyl conformations can bind to the receptor. Perhaps a variety of conformations of fentanyl can stabilize multiple conformations of the MOR, which can explain why fentanyl can induce different intracellular signaling and multiple physiological effects.

## Introduction

As of 2021, 51.6 million adults in the U.S. experience chronic pain, which is daily pain for at least three consecutive months. This has resulted in a loss of $685 billion to the U.S. economy in 2017^1−3^ due to death, high health care costs, and lost jobs. Opioids are widely administered to treat acute and chronic pain; however, the abuse of prescription and illicit opioids is a major health crisis in the United States.^4^ Opioids can be natural, semisynthetic, and synthetic chemicals that interact with the opioid receptors in the brain. The sap of the opium poppy plant (*Papaver somniferum*) is extracted to obtain morphinans, the backbone of many opioids.^5,6^ Morphinans are used for medicinal purposes or for the purpose of synthesizing heroin and other illegal narcotics. The leading cause of death by overdose in Europe and North America is attributed to not only prescription drugs, but also illicit intravenous drugs such as heroin, methadone, and fentanyl (*N*-phenyl-*N*-[1-(2-phenylethyl)piperidin-4-yl]propanamide).^4,7^ The current concern is that the clinical efficacy of these drugs are limited by the capacity to develop tolerance and addiction.^1,2,4,7^ Yet, opioids continue to be prescribed for their unrivaled ability to moderate severe pain.

The μ-opioid receptor (MOR) belongs to a subfamily of G-protein coupled receptors (GPCRs) which are widely studied due to their importance as therapeutic targets.^6,8−11^ The conformational flexibility that GPCRs have is vital to their ability to recognize ligands and activate or inactivate MOR.^12^ The activation of MOR due to an agonist binding involves specific conformational changes in the seven transmembrane helices (TM1-7), especially in highly conserved motifs (intramolecular switches).^8^ Specifically, the largest change in MOR activation is that the intracellular end of TM6 bends as much as 10 Å away from the helical core, whereas TM7 moves toward the core.^8^ TM7 and the intracellular halves of TM2, TM3, and TM6 rearrange to open a hydrophobic barrier that arranges with the W293^6.48^ rotamer toggle switch to form a water channel connecting the extracellular and intracellular sides.^9^ (Superscripts are residues in the Ballesteros–Weinstein numbering format.^13^)

Both beneficial and adverse effects of opioids are attributable to the binding of MOR agonists and are mediated by different signaling and regulatory pathways.^10,11^ The inhibitory heterotrimeric G-protein (G_i_) pathway is responsible for the beneficial effects of an MOR agonist, such as pain relief and euphoria, whereas the recruitment of β-arrestin (β_arr_) regulates the adverse effects, such as addiction, constipation, and especially respiratory depression.^12,14−18^ In contrast, MOR antagonists block the action of the agonist altogether. Experimental studies show that fentanyl and other unbiased opioids that signal through β_arr_ bind to helices 2, 3, 6, and 7 of MOR, whereas agonists that signal solely through G_i_ bind exclusively to helices 6 and 7. Thus, accurate prediction of the agonist-MOR binding is imperative for safe drug development.^10^ The ultimate goal of opioid research is to develop a novel MOR agonist that has selective bias for the G_i_ pathway.^19^

To better understand the structural basis for MOR function, Manglik et al. collected X-ray diffraction data from 25 crystals of the *Mus musculus*-OR-T4L (mMOR) protein bound to the irreversible morphinan antagonist β-funaltrexamine (β-FNA; PDB ID: 4DKL).^9,20,21^ β-FNA is a model ligand used to understand how antagonists interact with the inactive binding pocket of MOR. The morphinan core of β-FNA forms a salt-bridge with Asp147^3.32^ through its protonated amino group and a hydrogen bond between Tyr148^3.33^ and the endocyclic oxygen; these interactions are crucial requirements for ligands to bind to MOR.^8,22,23^ β-FNA also forms an irreversible covalent bond with the Lys233^5.39^ in the binding pocket of MOR, therefore it is not used clinically in humans.^24^ Our study aims to evaluate the conformational analysis of ligand structure through docking, so we opted to use naltrexone (*N*-cyclopropylmethylnoroxymorphone) instead of β-FNA since the computational docking program cannot reproduce covalent bonds. This opioid antagonist is identical to β-FNA, with the exception that the methyl-fumaramide group of β-FNA that forms the covalent bond with MOR is replaced with a ketone at the 6-position of naltrexone, so that the naltrexone ligand binds reversibly. Currently, naltrexone is used clinically in humans as a treatment for alcohol use disorder and opioid dependence.^25−27^

BU72 ((1*R*,2*S*,4*S*,5*S*,6*R*,9*R*,10*R*)-6-methoxy-5,20-dimethyl-4-phenyl-3,20-diazahexacyclo[8.7.3.1^5,9^.0^1,9^.0^2,6^.0^12,17^]henicosa-7,12(17),13,15-tetraen-15-ol) has a high affinity and extremely slow dissociation making it a good candidate for cocrystallization experiments with MOR, but it is too efficacious for human use.^28,29^ The first crystal structure of the MOR-BU72 complex had unexplained electron density around the BU72 ligand, and the BU72 ligand itself was in a highly strained conformation that deviated from ideal geometry.^8,30^ Munro’s reanalysis of the BU72-mMOR crystal structure revealed that changing the stereochemistry of the phenyl group from (*S*) to (*R*) resulted in a better fit to the electron density and a lower energy BU72 conformation.^31^ A second problem was unexplained electron density that connected BU72 with a histidine residue in the N-terminus. The short contacts and uninterrupted density were shown to be consistent with BU72 forming a covalent adduct with MOR, thus explaining the severe strain of the adduct.^30^ Because of the strain induced by the adduct and the nanobody used as a G protein substitute, the author concludes that this version of MOR will not be an accurate template for ligand docking and modeling to active G protein-bound MOR.^30^

Fentanyl is a strong β_arr_ biased agonist that elicits increased respiratory depression, the main side effect of β_arr_ signaling.^32^ Using cryo-electron microscopy (cryoEM), fentanyl has been experimentally determined in complex with human MOR (hMOR) bound to G_i_,^29^ so there is one experimental pose showing the binding site of fentanyl to hMOR. Previous molecular dynamics (MD) studies have provided insight into the binding modes of fentanyl, specifically its orientation, location, and interactions with amino acid residues. Podlewska et al. found that during MD simulations fentanyl’s mass center stayed in the same place, and only its flexible bonds rotated slightly throughout the simulations duration.^33^ However, other studies have found that fentanyl is able to move very deep into the binding pocket due to its linear nature and limited hydrogen bonding abilities to make important conformational changes that mediate β_arr_ signaling.^34^ Morphinan opioids, such as morphine, are unable to penetrate deeply into the pocket due to their structural rigidity and ability to make a stronger interaction with D147^3.32^. For example, an MD study found that, unlike morphine, fentanyl makes a contact with Y326^7.43^, which is located deep inside the binding pocket.^34^ In addition to interacting with Y326^7.43^, fentanyl’s aniline ring pushes residue M153^3.36^ downward to adopt a rotameric conformation which replaces W295^6.48^, an interaction that was not observed with a MOR-bound antagonist or morphine. An in vitro study demonstrated that this specific conformational shift of M153^3.36^ directly mediates β_arr_ signaling, but not G_i_ coupling.^35^ Although there are many computational studies of fentanyl,^10,33−37^ the exact nature of fentanyl’s binding mode remains unclear.

The benefit of using computational studies is that we can capture the structural diversity of the receptor and ligands that complement the data from experiments. Since protein binding pockets can change the conformation and p*K*_a_ of ligands once they enter the binding pocket, we have carried out a high-level quantum mechanical study to ascertain the conformations of the studied ligands as well as their charge states when in solution, prior to docking. Fentanyl and BU72 are clearly protonated in the experimental structures,^9,20,21^ but whether these ligands are protonated prior to entering the binding pocket is unknown. Therefore, p*K*_a_ calculations of each ligand in aqueous solution were completed. We then used Boltzmann calculations to estimate the concentrations of different conformations of each ligand at physiological temperature, and each conformer was docked into the MOR to establish whether there was room in the binding pocket for each. Based on previous knowledge of MOR-ligand interactions, we have used computational methods to gain insight into the bound and unbound ligand states. Computational methods illustrate specific steric and electronic features that must be present for opioid ligands to bind tightly to MOR. As demonstrated by the structure of fentanyl, certain ligands can be highly flexible and differ in their conformations, but still have the ability to bind tightly to MOR. Therefore, we will consider two hypotheses about ligand conformations: (1) the lowest free energy solution conformations fit into MOR without needing to rearrange, or (2) MOR itself and the ligands themselves adopt new conformations that optimize MOR-ligand binding. We also examine the impact of the bound intracellular protein on the conformation of MOR and its effect on ligand binding. This knowledge will be critical to developing agonist and antagonist models that build on those previously published, with the ultimate goal of novel treatments of opioid addiction and pain management to combat the current opioid crisis.

## Methods

### Aqueous-Phase Ligand Analysis

The computational process to explore the configurational space of the ligands BU72, naltrexone, and fentanyl followed a funnel method which first sampled a large region of conformational space, then used successively more accurate and more expensive calculations to end up with a small set of conformations of the lowest free energy structures. All calculations of ligands were computed using the universal solvation model density (SMD)^38^ based on solute electron density to produce structures that are optimized in aqueous solution modeled using the CREST (Conformer-Rotamer-Ensemble Sampling Tool)^39^ conformational sampling routine with the semiempirical GFN2-*x*TB method.^40,41^ This algorithm takes an initial pool of randomly generated configurations and updates them according to an evolutionary algorithm, finishing with a final set of converged structures. The initial pool size was chosen to be 1000 configurations, and the process ran for 20,000 iterations or until convergence. The final set of GFN2 structures were then screened for uniqueness to eliminate double counting by comparing their rotational constants and energies. Two structures were deemed identical if their rotational constants and energies were within 1% or 0.1 kcal·mol^–1^ of one another, respectively. The CREST step generated 243 GFN2 unique conformers of fentanyl, 47 GFN2 conformers of naltrexone, and 8 GFN2 conformers of BU72. These geometries generated in the GA step were then refined using ωB97X-D^42,43^ density functional theory (DFT) geometry optimizations^44,45^ with the 6-31++G** basis set,^46−50^ using Gaussian 2016.^51^ Geometries were optimized using tight convergence criteria (RMS Force 0.000010 au, maximum displacement = 0.000060 au), and the harmonic vibrational frequencies were calculated on the converged geometries. The final set of DFT structures were screened for uniqueness, resulting in 87 unique fentanyl conformers, 27 unique naltrexone conformers, and three BU72 conformers. In order to assess the thermodynamic stability of these conformers, the ωB97X-D harmonic vibrational frequencies were scaled by 0.971 and thermodynamic corrections for *H*_corr_, *S*_corr_, and *G*_corr_ at 310.15 K were computed using the THERMO.pl script from the National Institute of Science and Technology.^52^

In order to improve the accuracy of the electronic energies, the domain local-pair natural-orbital (DLPNO) couple-cluster methods with single, double, and perturbative triple excitations (CCSD(T)) were employed.^53−66^ CCSD(T) calculations with large basis sets or by extrapolation to the complete basis set (CBS) limit are the “gold standard” for ground-state quantum chemistry, with uncertainties less than 1 kcal·mol^–1^.^67,68^ The electronic energies of the final sets of DFT conformations were calculated at the DLPNO-CCSD(T)-cc-pVnZ//ωB97X-D/6-31++G** level of theory^53−66^ implemented in the ORCA 5.0.1 program.^69^ In-house scripts were used to compute the CBS extrapolations using the double-ζ, triple-ζ, and quadruple-ζ basis sets (n = D, T, Q)^70−72^ using a 4-5 inverse polynomial CBS extrapolation scheme.^73^ Lastly, these high-level electronic energies were combined with the DFT thermodynamic values to compute the Gibbs free energy values for every DFT geometry. All final structures were compared against the lowest energy structures with both functionals using ArbAlign,^74^ yielding root-mean-square deviations (RMSDs) that were then visualized graphically using Avogadro and Chimera.^75,76^ Expected populations based on Boltzmann calculations at physiological temperature were used to estimate the abundance of different conformers of a particular ligand in aqueous solution, and are presented in the Figures.

#### p*K*_a_ Calculation of BU72

The acid dissociation constant, or p*K*_a_, is an essential part of understanding many chemistry and biochemistry reactions. We computed the p*K*_a_, of the BU72 ligand to determine (1) if it will be protonated at physiological pH, (2) if this occurs prior to binding to MOR, and (3) and to quantify the Gibbs free energy difference between the protonated ligand and the neutral ligand. By calculating the p*K*_a_, we gain insight into the relative concentration of opioid ligands that are charged in solution as well as which atom in a particular molecule is charged, and how that impacts the ligand-MOR binding energy. The dissociation reaction is defined as in eq 1
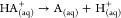
1and the p*K*_a_ for this reaction is defined in eq 2
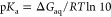
2where HA^+^ is the acid or dissociating molecule of interest and A is the conjugate base,^77−79^*R* is the ideal gas constant, *T* is the temperature, and Δ*G*_aq_ is the change of the Gibbs free energy in aqueous solution. For ligands whose p*K*_a_ values have not been determined experimentally, computing them is useful. It should be noted that when calculating p*K*_a_, it is important that Gibbs free energy calculations are computed as accurately as possible, as an error of 1.36 kcal·mol^–1^ in the free energy of reaction 1 results in an error of 1 p*K*_a_ unit in reaction 2.^77,79,80^

**Figure 1:**
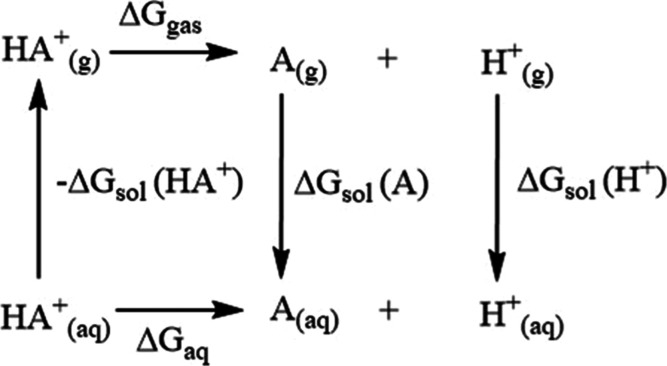
Proton-based thermodynamic cycle. Reproduced from ref (81). Copyright 2001 American Chemical Society.

Thermodynamic cycles are used to calculate p*K*_a_ by determining Δ*G*_aq_, the free energy in solution, from the free energy of dissociation in the gas phase and the free energies of solvation of HA^+^, A, and H^+^ as seen in Figure 1.^77,79^

There are three different methods used to calculate thermodynamic cycles: (1) using gas phase geometries for HA^+^ and A in the entire cycle, (2) using solution phase geometries for HA^+^ and A in the cycle, and (3) using the gas phase geometries for *ΔG*_gas_ and solution phase geometries for the free energy of solvation (*ΔG*_sol_). We used method 2. We used *ΔG*_sol_ along with the gas phase and aqueous phase free energies to determine p*K*_a_ using the following equations, 3–5:
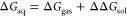
3where
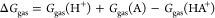
4and
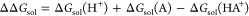
5All of these free energy values are determined computationally except *ΔG*_sol_(H^+^) and *G*_gas_(H^+^). A proton has no electrons; therefore, its free energy cannot be calculated quantum mechanically. These values have been determined experimentally to be *ΔG*_sol_(H^+^) = −265.6 kcal·mol^–1^ and *G*_gas_(H^+^) = 6.28 kcal·mol^–1^ at 298 K.^79^ The Gibbs free energy of the proton can also be calculated from the Sackur–Tetrode equation.^77^ The largest uncertainty is for the value of *ΔG*_sol_(H^+^).^77^

#### Structural Analysis of mMOR-Nanobody and hMOR-G_i_

The coordinates of the experimental hMOR-G_i_ and mMOR-nanobody complexes (PDB IDs 8EF5(10) and 5C1M,^8^ respectively) were obtained from the Protein Data Bank.^82,83^ RMSD analysis of these structures was performed with the visual molecular dynamics program version 1.9.4 (VMD).^84^ The RMSD Trajectory tool plugin in VMD for all the non-hydrogen protein backbone atoms as shown in eq 6
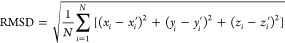
6With *x*_*i*_ and *x*_*i*_^′^, *y*_*i*_ and *y*_*i*_^′^, and *z*_*i*_ and *z*_*i*_^′^ are the respective *x*-, *y*-, and *z*-coordinates of the *i*th atom for *N* total atoms within two molecules.

The RMSDs of the entire receptor and the individual helices backbone atoms were calculated based on the sequence numbering. The hMOR has two more residues than mMOR, so ensure that the conserved residues had matching numbers, the residue numbering of the mMOR was increased by two. The conserved residues of the receptor are #66 to 349. The seven transmembrane (TM) helices were denoted by the following ranges of residues: TM1: 66 to 98, TM2: 103 to 133, TM3: 138 to 173, TM4: 182 to 207, TM5: 226 to 264, TM6: 270 to 308, and TM7: 313 to 338. All residue numbers correspond to those in hMOR, and the numbering of the helical portions of the receptor are given by the PDB structure of 8EF5.^10^

#### Principal Component Analysis

The Bio3D package^85^ was used to perform principal component analysis (PCA) on a set of experimentally determined MOR structures to identify the significant structural differences between the active and inactive conformations using procedures outlined previously.^85,86^ A basic local alignment search tool (BLAST) search^87^ of the Protein Data Bank^82^ was performed with the input target sequence of the receptor (“*R*” chain) of the MOR in complex with fentanyl and the G_i_ (PDB ID: 8EF5).^10^ Five hundred and forty-nine sequences were identified. These hits were filtered with a cutoff value of 322 corresponding to the negative log of the *E*-value of the BLAST results. Eleven sequences remained, all of which are the receptor chains of MORs, which ranged from 100 to 93.9% sequence identity with the target sequence. The target sequence was added to the 11 from the BLAST search for a total of 12 sequences to be used for subsequent analysis. The sequences were aligned using the Clustal Omega program^88^ and a structural invariant core was determined and used to superimpose the 12 aligned structures. The structures were clustered into four groups based on their α-carbon atom RMSDs of the superimposed structures. Gaps in the sequences were removed, and PCA was performed on the Cartesian coordinates of the structure’s atoms. Eight hundred and twenty-eight eigenvalues were identified, and the proportion of the variances for each PC was calculated. The variance represents the atomic motion in each direction and corresponds to an eigenvalue.^85^ PCs 1 and 2 accounted for 86.6% of the cumulative variance with PC1 accounting for 71.0% and PC2 accounting for 15.6% of their respective variances.

#### Docking of Ligands to the μ-Opioid Receptor

##### Protein Preparation

The protein structures of the mMOR bound to the antagonist β-FNA, mMOR-nanobody, and hMOR-G_i_ (PDB IDs: 4DKL, 5C1M, and 8EF5) were created using Protein Preparation Wizard as part of the Schrodinger 2023-2 software suite (Schrödinger release 2023-2: Jaguar, Schrödinger, LLC, New York, NY, 2023). This program assigns bond orders using the CCD database, adds hydrogens, creates disulfide bonds, fills in missing side-chains using the Prime program,^89,90^ and generates Epik states for the heteroatoms. PROPKA^91^ was used to calculate the p*K*_a_ of each titratable amino acid at 7.4 which created charged amino acids. Hydrogen bonds were optimized by sampling different amino acid rotamers and removing overlap between hydrogens. Heavy atoms were minimized to a convergence RMSD of 0.30 Å. The force field used was OPLS4.^92^

##### Ligand Atomic Charge Determination

To determine the atomic charges for the ligand, single point energy calculations were performed using the Jaguar application,^93^ which is part of the Schrodinger 2023-2 software suite. The ligands were solvated with the Poisson–Boltzmann finite elements (PBF) model.^94−96^ The partial charges were determined by calculating the Mulliken populations using DFT with automatic self-consistent field (SCF) spin treatment for open and closed shell systems^97^ based on the respective ligand’s multiplicity, a nonrelativistic Hamiltonian, and medium grid density.^98^ The computation used the B3LYP-D3 theory^99−102^ and the 6-31G** basis set.^103^ The SCF used quick accuracy^104^ with 48 maximum iterations^105^ to reach a convergence^106^ with an energy change of 5 × 10^–5^ Hartree and an RMS density matrix change of 5 × 10^–6^ using the DIIS convergence scheme.^107^

##### Glide Grid Generation

To orient the ligands during docking, a grid of the docking site surface was generated for each receptor using the Receptor Grid Generation program, which is part of the Glide program^108,109^ in the Schrodinger 2023-2 suite (Schrödinger release 2023-2: Glide, Schrödinger, LLC, New York, NY, 2023). The ligands were docked into a generated box of length 20 Å and were confined to a smaller box with a length of 10 Å. The center of the box was calculated based on specified residues in the binding site. For the fentanyl-bound MOR (PDB ID: 8EF5), the centroid of the following residues was used: N129^2.63^, W135^EC1^, D149^3.32^, C219^EC2^, E231^5.36^, K235^5.40^, W295^6.48^, I324^7.38^, and Y338^7.42^. In the BU72-bound MOR (PDB ID: 5C1M), the binding site residues used were D147^3.32^ and Y148^3.33^.

##### Glide Docking

The Glide docking program, which is part of the Schrodinger 2023-2 suite was used to determine the ligand position in the receptors. Each ligand with was docked rigidly using extra precision mode. Van der Waals radii of the nonpolar receptor atoms were multiplied by a scaling factor of 0.80 to decrease the penalties of close contacts. Nonpolar atoms are defined as having a partial charge of 0.15 or less. Ring conformations were discarded if their energy was greater than 2.5 kcal·mol^–1^ above the lowest energy conformation. The input structure was regenerated based on the connectivity, bond orders, and stereochemistry. After the initial phase of docking, 5000 poses of each ligand within 100 kcal·mol^–1^ of the lowest energy pose were kept for the next stage. Eight hundred ligand poses with the best energy were minimized with the OPLS3^110^ nonbonded interaction grid. For minimization, the distance-dependent dielectric constant was set at 2.0, and the maximum number of minimization steps was 100. If a ligand pose had a GlideScore greater than 0.50 kcal·mol^–1^, it was rejected. The ten ligand poses with the best energies were saved for post-docking full force-field minimization. Strain correction terms that apply penalties for high strains were added to the GlideScore if they were above the value of 4.0. The scaling factor for excess strain energy, which is multiplied by the Van der Waals radii of nonpolar receptor atoms, was 0.25. Poses with Coulomb–Van der Waals energies between the ligand and receptor more positive than 0.0 kcal·mol^–1^ were rejected. Ligand poses that had an RMSD less than 0.5 Å, with a maximum atomic displacement less than 1.3 Å, or with hydroxyl and thiol hydrogens torsional within 40° were considered to be duplicates and were discarded. Ligand poses were sorted according to best docking score. Docking scores are based on rudimentary metrics, so the calculated energies should be used to understand relative trends and are not at the same level as the relative Gibbs free energies for the ligands that are produced using high level quantum chemistry.

## Results and Discussion

### Mouse (mMOR-Nanobody) and Human (hMOR-G_i_) Structure Comparison

**Figure 2:**
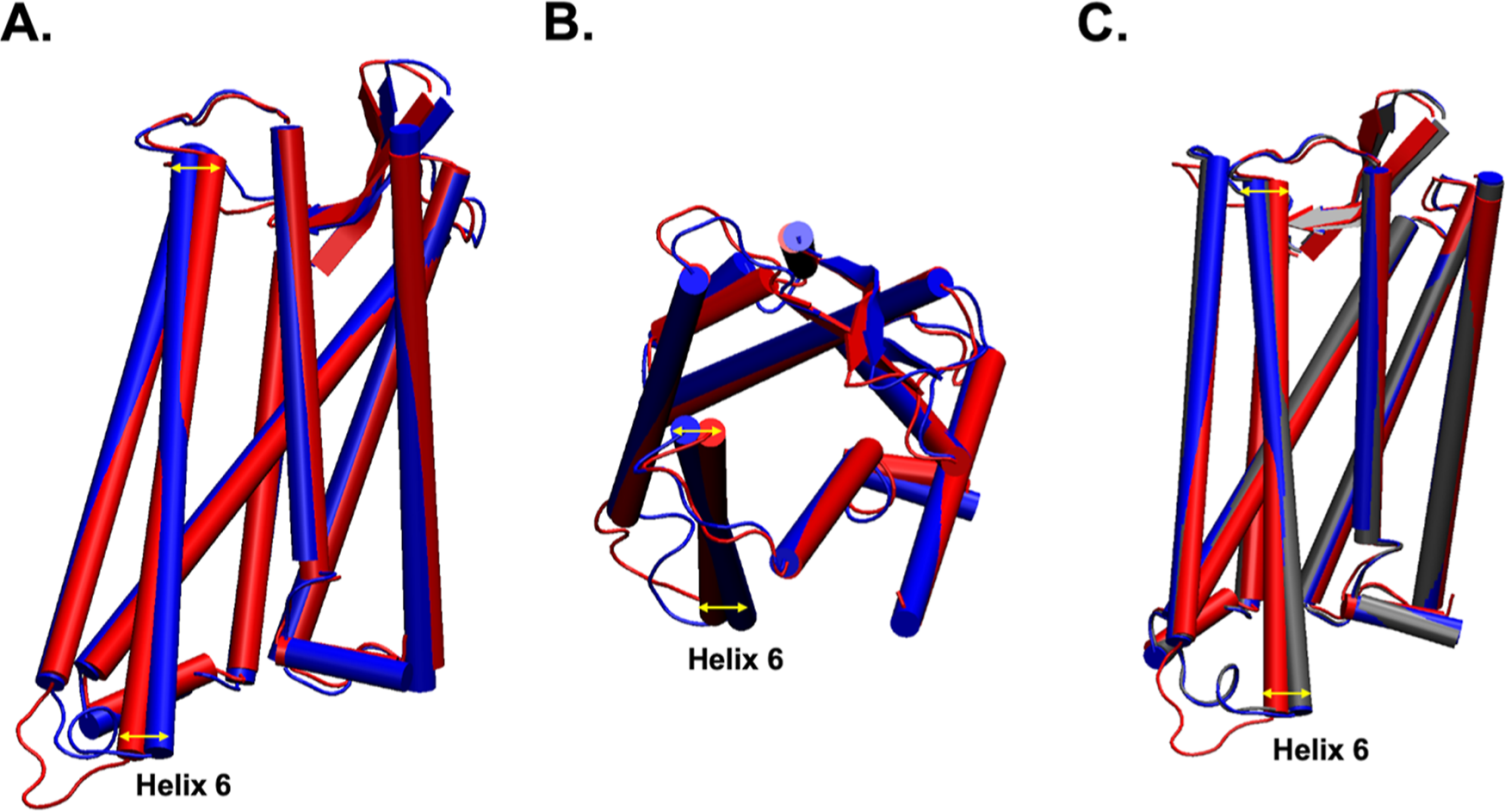
(A) Side view and (B) extracellular view of the alignment of fentanyl-bound hMOR-G_i_ (blue, PDB ID: 8EF5)^10^ with BU72-bound mMOR-nanobody (red, PDB ID: 5C1M),^8^ and (C) morphine-bound hMOR-G_i_ (gray, PDB ID: 8EF6).^10^ Yellow arrows indicate the displacement of helix 6 (indicated) between the structures.

**Figure 3:**
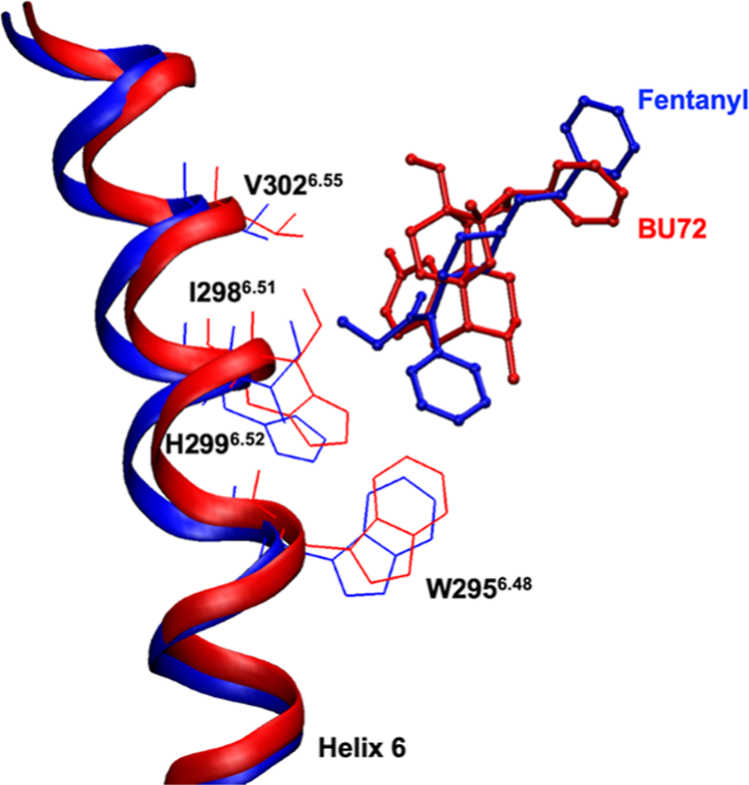
Comparison of the helix 6 fentanyl-binding residues in fentanyl-bound hMOR-G_i_ (blue, PDB ID: 8EF5)^10^ and BU72-bound mMOR-nanobody (red, PDB ID: 5C1M).^8^ Amino acids W295^6.48^, I298^6.51^, H299^6.52^, and V302^6.55^ (line representation) and bound ligands, fentanyl and BU72 (blue and red, respectively, ball-and-stick side chains representation), are indicated.

The mMOR-nanobody in complex with BU72^8^ exhibits a different structure than hMOR-G_i_ in complex with fentanyl.^10^ As discussed in previous studies, comparison of these experimental structures with respect to the inactive antagonist β-FNA-bound mMOR^20^ indicate that there are similar conformational changes upon activation.^10^ However, the RMSD of the protein backbone of the conserved residues (hMOR sequence #S66-C349) of mMOR-nanobody and hMOR-G_i_ is 1.372 Å. Substantial differences occur in helices 5 and 6, which are displayed in Figure 2A,B. The RMSDs of the backbones of the individual helices range from 0.579 Å for helix 2 to 1.683 Å for helix 6. Helices 6 and 7 have been hypothesized as being important for distinguishing biased ligands in MOR. Ligands that induce unbiased signaling interact with helices 6 and 7 whereas ligands with reduced interactions with helices 6 and 7 signal only through the G_i_ pathway,^10^ so finding the correct position for helix 6 is extremely important for predicting accurate ligand poses. Fentanyl is an unbiased opioid, so it has interactions with residues on helix 6 including W295^6.48^, I298^6.51^, H299^6.52^, and V302^6.55^.^10^ The RMSD of the backbones of these specific residues in hMOR-G_i_ is 1.205 Å with respect to those in the mMOR-nanobody model. In comparison, the RMSD for all the backbone residues in the fentanyl binding site is 0.851 Å. Figure 3 shows a comparison of these helix 6 fentanyl-binding residues in the hMOR-G_i_ and mMOR-nanobody structures. The fentanyl ligand in the hMOR-G_i_ clashes with the residues in the mMOR-nanobody structure explaining why the extracellular end of helix 6 sweeps outward in the hMOR-G_i_ structure.

**Figure 4:**
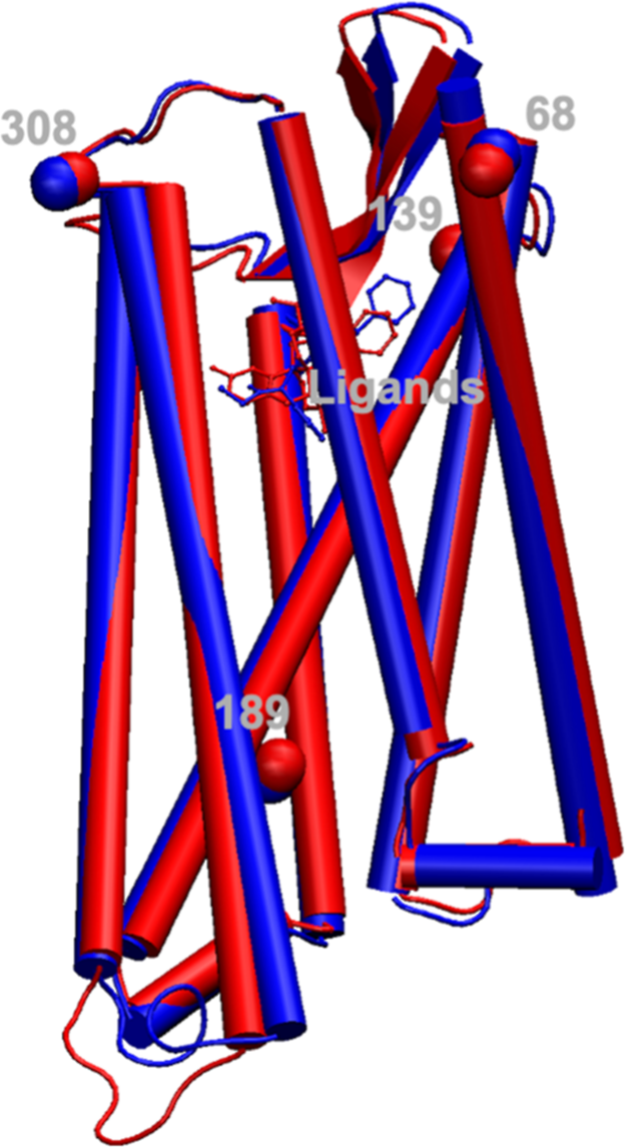
Nonconserved residues (van der Waals representation of the α-carbon atoms) between fentanyl-bound hMOR-G_i_ (blue, PDB ID: 8EF5)^10^ and BU72-bound mMOR-nanobody (red, PDB ID: 5C1M)^8^ are shown in relation to the respective ligands (ball-and-stick representation).

One hypothesis for the difference in the structures is the difference in the sequences. The conserved regions of the two sequences (hMOR sequence #S66-C349) were aligned using BLAST software, and the sequence identity was 99%. There were only four residues, I68^N-terminus^, T139^EC1^, I189^4.45^, and V308^EC3^, that were not conserved. None of these residues are in the binding site or interact with the ligand, nanobody, or G_i_ as shown in Figure 4, so these residues are not likely to have a significant impact on the global conformation of the receptor, negating that hypothesis. Another hypothesis is that the ligands, fentanyl and BU72, stabilize different conformations of MOR. However, BU72 is a morphinan agonist, and the backbone RMSD of the fentanyl-bound receptor versus the morphine-bound receptor is 0.352 Å. The binding site of fentanyl in the morphine-bound hMOR-G_i_ conformation has an RMSD of 0.390 Å with respect to the fentanyl-bound hMOR-G_i_ conformation, which is lower than that of the site in mMOR-nanobody with an RMSD of 0.870 Å. The RMSD of the morphine-binding site in the fentanyl-bound hMOR-G_i_ complex is 0.435 Å, which is smaller than that of the RMSD of the same site in the mMOR-nanobody complex, which is 0.752 Å. Given the structural similarity of morphine and BU72, it is expected that they stabilize similar binding site conformations. However, 91% of the residues in the BU72 binding site^8^ are found in the fentanyl binding site and in the morphine binding site of hMOR, so the different ligands bind to the same site of the receptor. Thus, this infers that the nanobody stabilizes a different conformation of MOR than G_i_ does.

### Analysis of Experimentally Determined MOR Structures

**Figure 5:**
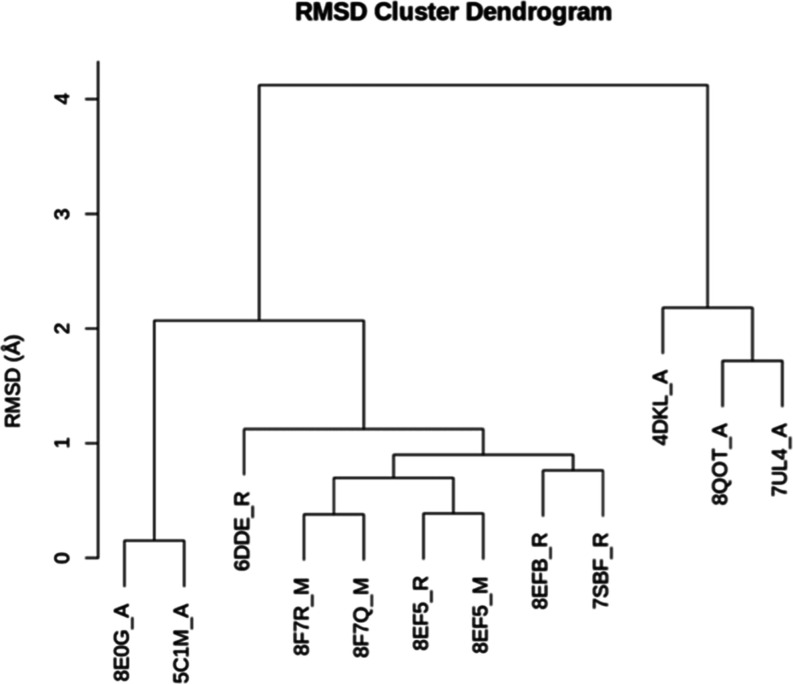
RMSD cluster dendrogram for 12 MORs that are indicated by their respective PDB IDs and receptor chain name.

To determine the similarity of nanobody-bound mMOR and the G_i_-bound hMOR, a BLAST search was performed that identified 11 experimentally determined structures of the MOR. An RMSD analysis was performed on these 11 plus the receptor of the G_i_-bound hMOR (chain R of PDB ID 8EF5).^10^Figure 5 shows the dendrogram of clustering the 12 structures by RMSD. The nine “activated” structures that are bound to agonists are clustered in one branch whereas the three “inactive” structures that are bound to antagonists are in a separate branch, indicating significant structural differences as expected. The RMSDs between the active and inactive structures range from 3.428 to 4.121 Å. Within the activated structures, there are two distinct branches—one consisting of MORs bound to the G_i_ (PDB IDs: 6DDE, 8F7R, 8F7Q, 8EF5, 8EFB, and 7SBF)^10,11,23,111^ and one consisting of MORs bound to a nanobody (PDB IDs: 8E0G and 5C1M)^8,30^ with RMSDs ranging from 1.683 to 2.071 Å. This shows that the nanobody and G_i_ proteins consistently stabilize different conformations of the MOR.

**Figure 6:**
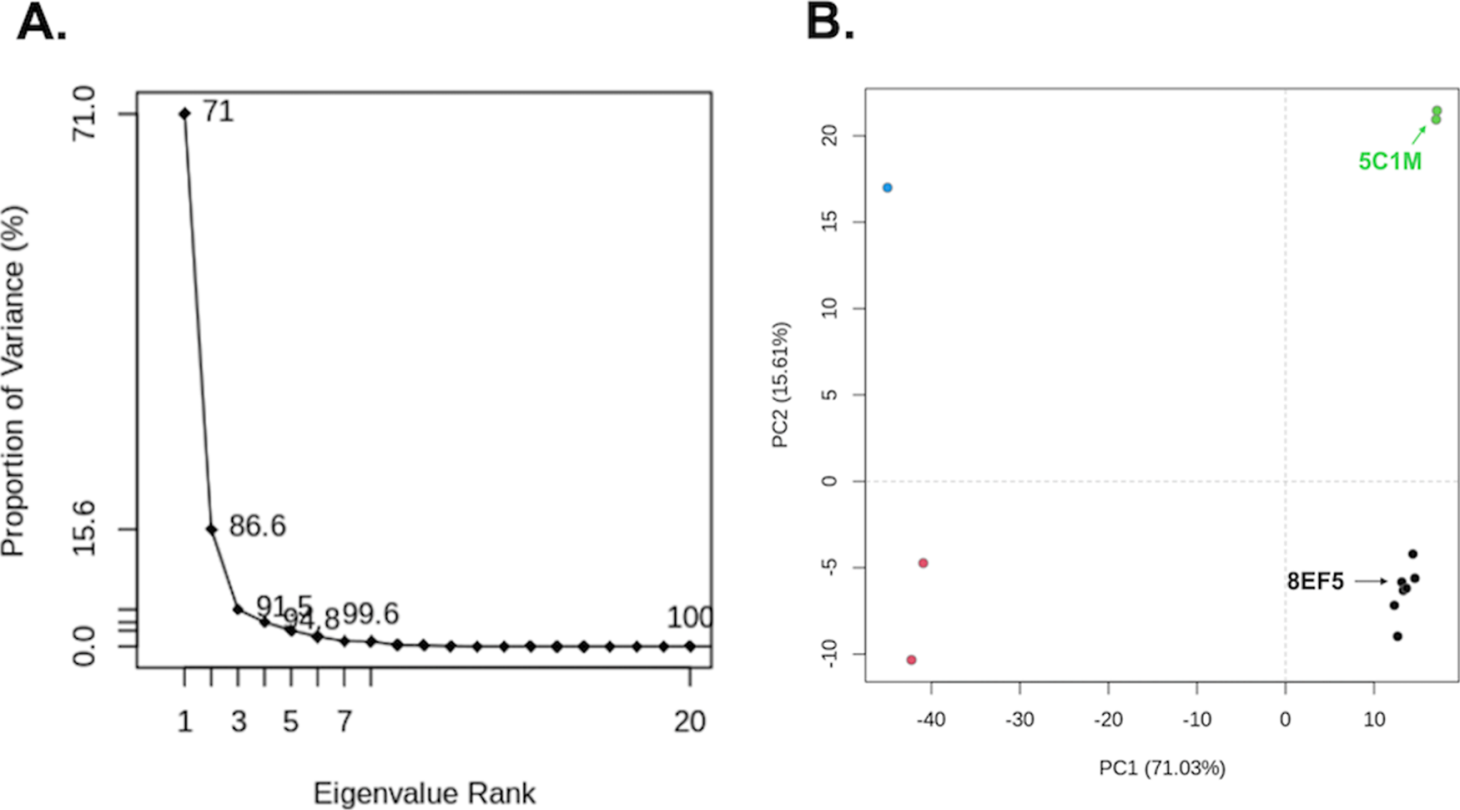
PCA analysis of 12 experimentally determined MORs. (A) Proportion of variance versus eigenvalue rank for 20 PCs. (B) PC1 vs PC2 for the MORs that have been clustered into four groups that are represented by different colors—blue (chain A of PDB ID 4DKL),^20^ red (chain A of PDB ID 8QOT(122) and chain A of PDB ID 7UL4(123)), green (chain A of PDB ID 8E0G(30) and chain A of PDB ID 5C1M(8)), and black (chain R of PDB ID 6DDE,^23^ chain M of PDB ID: 8F7R,^111^ chain M of PDB ID: 8F7Q,^111^ chains M and R of PDB ID: 8EF5,^10^ chain R of PDB ID: 8EFB,^10^ chain R of PDB: 7SBF.^11^ Chain A of PDB ID 5C1M and chain R of PDB ID 8EF5 are labeled and indicated by arrows.

**Figure 7:**
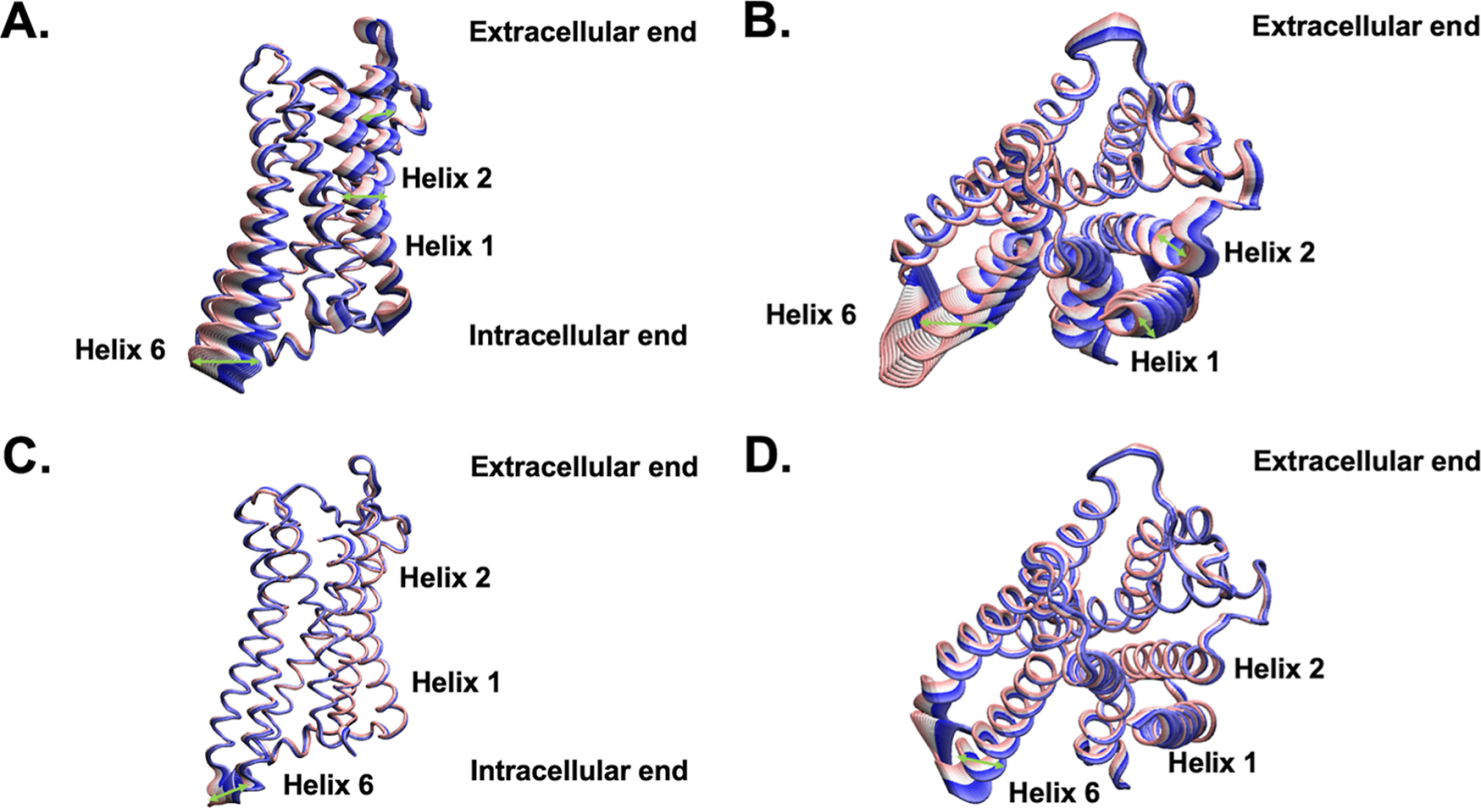
Visualization of the principal components (PCs) of 12 experimentally determined MORs. (A) Side and (B) extracellular view of PC1. (C) Side and (D) extracellular view of PC2. The extracellular and intracellular ends of the receptor are indicated. Helices 1, 2, and 6 are labeled. Green arrows indicate the movement of the helices.

To further characterize the structural differences between the multiple activation states of MOR, PCA was performed of the 12 experimentally determined receptor structures. The objective of PCA is to show that the activated experimental structures, particularly PDB IDs 5C1M and 8EF5, are structurally distinct from one another and should not considered to be equivalent. In previous studies, Bio3D’s PCA application has analyzed the experimental structures of other proteins including S100A1,^86^ Gα,^112^ and kinesin.^113^ In all three cases, PCA identified structural differences between various categories of experimental structures based on sequence identity, the type of bound cofactors, and/or activation states. PCA has also been used to characterize the differences between the MD trajectories of A-type, B-type, and PNA-type DNA triple helices^114^ and A-type, B-type, and PNA-type PNA-DNA-PNA triple helices.^115^ Here, these same techniques were applied to the 12 MORs to show their structural relationships in PC1 and PC2 space. The proportion of variance for each eigenvalue are shown in Figure 6A. PC1 has a proportion of variance of 71.03%. PC2, on the other hand, has a proportion of variance of 15.61%. Therefore, the first two PCs encapsulate approximately 86% of the variance of the atomic movement and can provide a thorough summary of the conformational space sampled by the MOR experimental structures. The 12 structures were clustered into four groups based on their RMSDs and projected onto PC1 versus PC2 space in Figure 6B. There is a clear distinction between the active and inactive structures in PC1 space. The antagonist-bound mMOR (PDB ID: 4DKL) has a PC1 of −45.0, where the inactive nanobody-bound structures (PDB IDs: 7UL4 and 8QOT) have PC1s of −42.2 and −40.9, respectively. In comparison, the agonist-bound structures have PC1s ranging from 12.7 to 17.1. PC1 is visualized in Figure 7A,B. PC1 is characterized by the movement of the intracellular end of helix 6 moving away from the center of the helical bundle. This corresponds to the activation mechanism as the intracellular end is widening to allow the intracellular G_i_ to bind. Similar movements in helix 6 have been observed in the activation mechanism of other class A GPCRs.^10,116−121^ Therefore, PCA can distinguish the clear structural differences between the active and inactive forms of the MORs.

While PC2 accounts for a much smaller percentage of the variance of the RMSD, it still distinguishes between the clusters of MORs. The inactive structures are grouped into two clusters that are separated in PC2 space. PDB ID 4DKL is a mMOR bound only to an antagonist in the orthosteric binding site^20^ and is separated by the group containing PDB IDs 7UL4 and 8QOT that are both mMORs bound to nanobodies. 4DKL has a PC2 of 17.0, whereas 7UL4 has a PC2 of −10.3 and 8QOT has a PC2 of −4.7. Similarly, the activated structures of MOR are also separated in PC2 space depending on the type of bound intracellular protein. PDB IDs 5C1M and 8E0G are both bound to BU72 and a nanobody and are clustered into one group with PC2s of 20.9 and 21.5, respectively. The remaining structures (PDB IDs 6DDE, 8F7R, 8F7Q, 8EF5 chains R and M, 8EFB, and 7SBF)^10,11,23,111^ are all bound to agonists and G_i_. They are clustered in a separate group with PC2s ranging from −4.2 to −9.0. Figure 7A,B visualize PC1 and Figure 7B and C visualize PC2. Most of the movement in PC2 occurs in the third intracellular loop (IC3). This can be explained as the clusters in PC2 space are differentiated by the types of intracellular proteins that the receptors bind to and also how they interact with IC3. Movement in the IC3 causes slight differences in helix 6 as it is connected to IC3. Since 8EF5 and 5C1M are clustered into separate groups separated in PC2 space, it is confirmed that despite both receptor structures are considered to be activated because they are bound to agonists, they are structurally different and are not comparable, which will impact the binding of ligands to the orthosteric site.

### Analysis of Fentanyl Solution Structures

**Figure 8:**
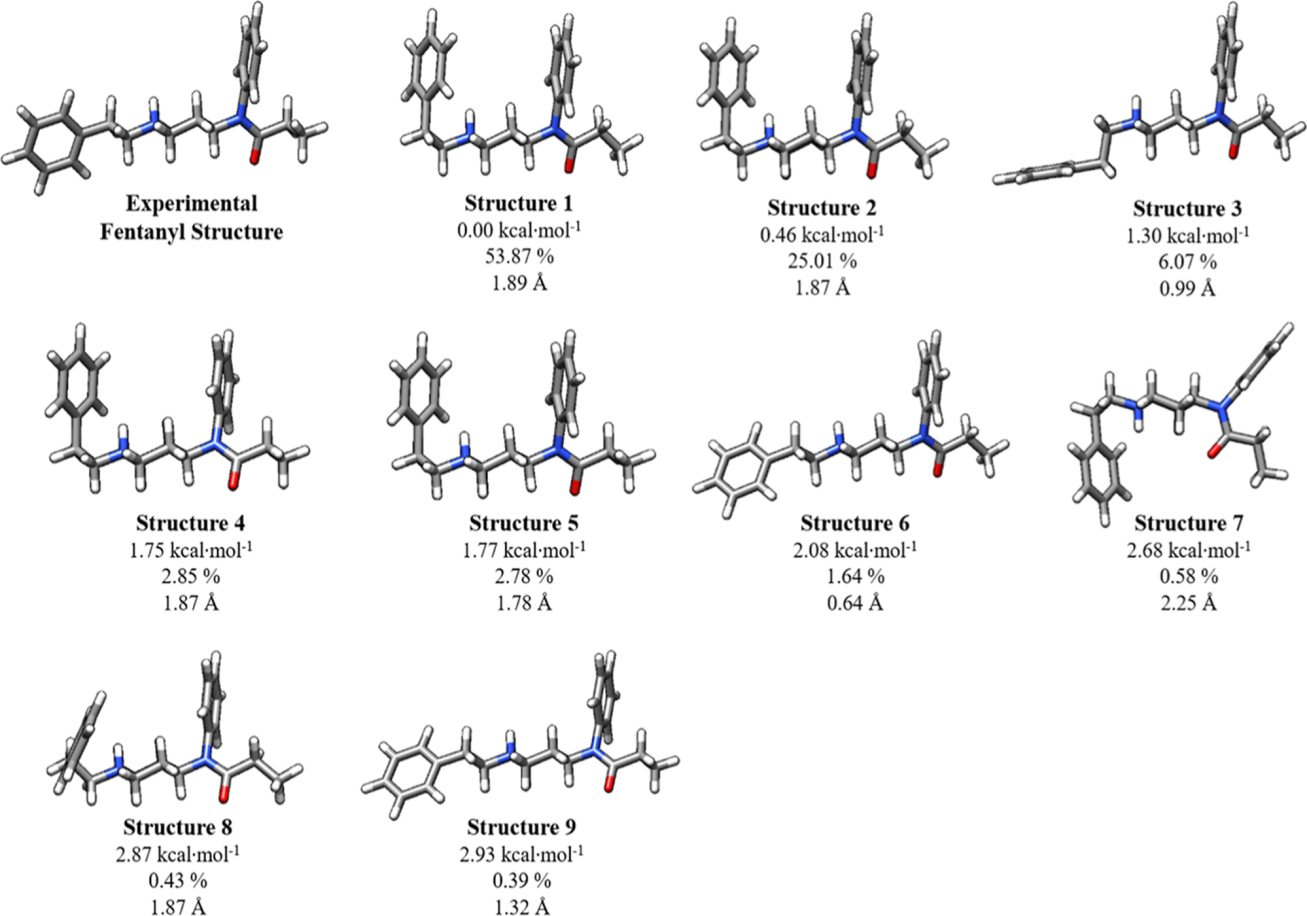
Lowest energy conformations of fentanyl at 310.15 K in aqueous solution generated at the ωB97X-D/6-31++G**/SMD level of theory. Structures are ordered in terms of Gibbs free energy relative to the local minimum energy structure computed at the DLPNO-CCSD(T)/CBS/SMD//ωB97X-D/6-31++G**/SMD level of theory. Relative Gibbs free energies (Δ*G*°) with respect to the minimum Gibbs free energy structure are shown in kcal·mol^–1^. Reported below each Δ*G*° are the percent abundance of each structure in aqueous solution at 310.15 K calculated using the Boltzmann equation. The RMSD of the heavy atoms for each structure in comparison to the fentanyl experimental structure (PDB ID: 8EF5)^10^ is included.

Ultimately, the ligand binding has an impact on the MOR structure. To understand if the ligand conformation was the same or different in solution relative to the MOR-bound structure, the lowest energy conformations of fentanyl at 310.15 K were determined using the DLPNO-CCSD(T)/CBS/SMD//ωB97X-D/6-31++G**/SMD model chemistry. These high-level quantum chemistry calculations on solution conformations are compared to the conformation of fentanyl when bound to hMOR.^10^ Structures are ordered in terms of relative Gibbs free energies (Δ*G*°) with respect to the minimum Gibbs free energy structure, structure 1. Structures 3, 6, and 9 in Figure 8 resemble the experimental structure. Boltzmann calculations predict that all these structures are available in aqueous solution at 310.15 K because they are within 3 kcal·mol^–1^ of the minimum. The law of mass action dictates that when one conformer binds to MOR, it will leave the solution, and the resulting new equilibrium concentrations will result in more conformers of the ligand that is bound to the MOR. Thus, if structure 6, which is the closest conformation to the experimental structure (RMSD = 0.63 Å), were to bind to MOR, that conformation would be repopulated as other conformations shift toward structure 6. Note that only small conformational changes are required for structures 3, 6, and 9, which make up 8% of the conformers at 310.15 K, to reproduce the conformation of the experimental structure. Fentanyl has high conformational flexibility, with at least nine conformers present in solution.

### Analysis of the BU72 Solution Structures

**Figure 9:**
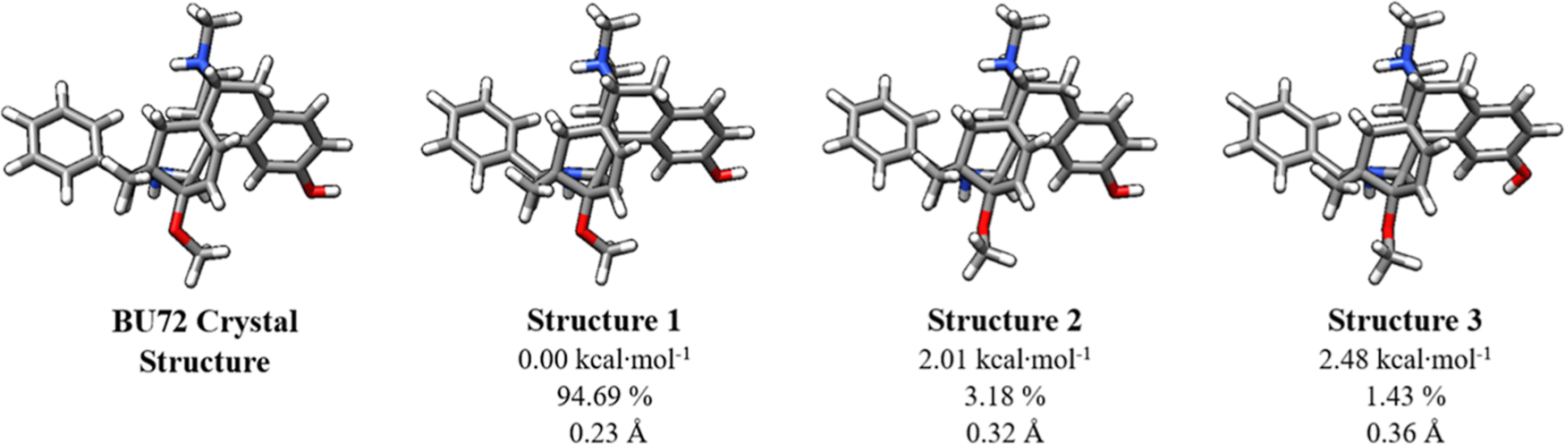
Lowest energy conformations of BU72 at 310.15 K in aqueous solution generated at the ωB97X-D/6-31++G**/SMD level of theory. Structures are ordered in terms of Gibbs free energy (Δ*G*°) relative to the local minimum energy structure computed at the DLPNO-CCSD(T)/CBS/SMD//ωB97X-D/6-31++G**/SMD level of theory. Relative Gibbs free energies are shown in kcal·mol^–1^. Reported below each Δ*G*° are the percent abundance of each structure in aqueous solution at 310.15 K calculated using the Boltzmann equation. The RMSD of the heavy atoms for each structure in comparison to the BU72 crystal structure (PDB ID: 5C1M)^8^ is included.

BU72 has much less conformational flexibility than fentanyl because of its constrained structure as shown in Figure 9. The only differences between the three solution structures lies in the rotation of the OCH_3_ and OH groups. The p*K*_a_’s of the nitrogen on fentanyl^124^ (8.43) and naltrexone^125^ (8.38) are known, in the 8.4 range, but the p*K*_a_ of BU72 has not been reported. Given the controversy in the BU72 crystal structure,^30^ we felt that it was important to calculate the p*K*_a_ of the nitrogen that would make the same salt-bridge with the mMOR Asp147^3.32^ residue as β-FNA.^20^ We calculated the p*K*_a_ of the nitrogen on structure 1 of BU72 that would make this same ionic interaction as naltrexone (since it is so similar to β-FNA) and predict a value of 9.2 at 310.15 K. This value is quite similar to those for fentanyl and naltrexone, and our methods should be accurate to within a p*K*_a_ unit,^77−81,126^ so it is highly probable that BU72 is protonated at the equivalent nitrogen. All three BU72 structures depicted in Figure 9 are quite close in conformation, and nearly identical to the crystal structure with a RMSD range of 0.23–0.36 Å. The main difference is that in the crystal structure, the protonated nitrogen that should be tetrahedral, is flattened out in the crystal structure,^8^ which may be because BU72 is covalently bound to MOR or some other error in the crystallographic electron density map.^30^

### Analysis of Naltrexone Solution Structures

**Figure 10:**
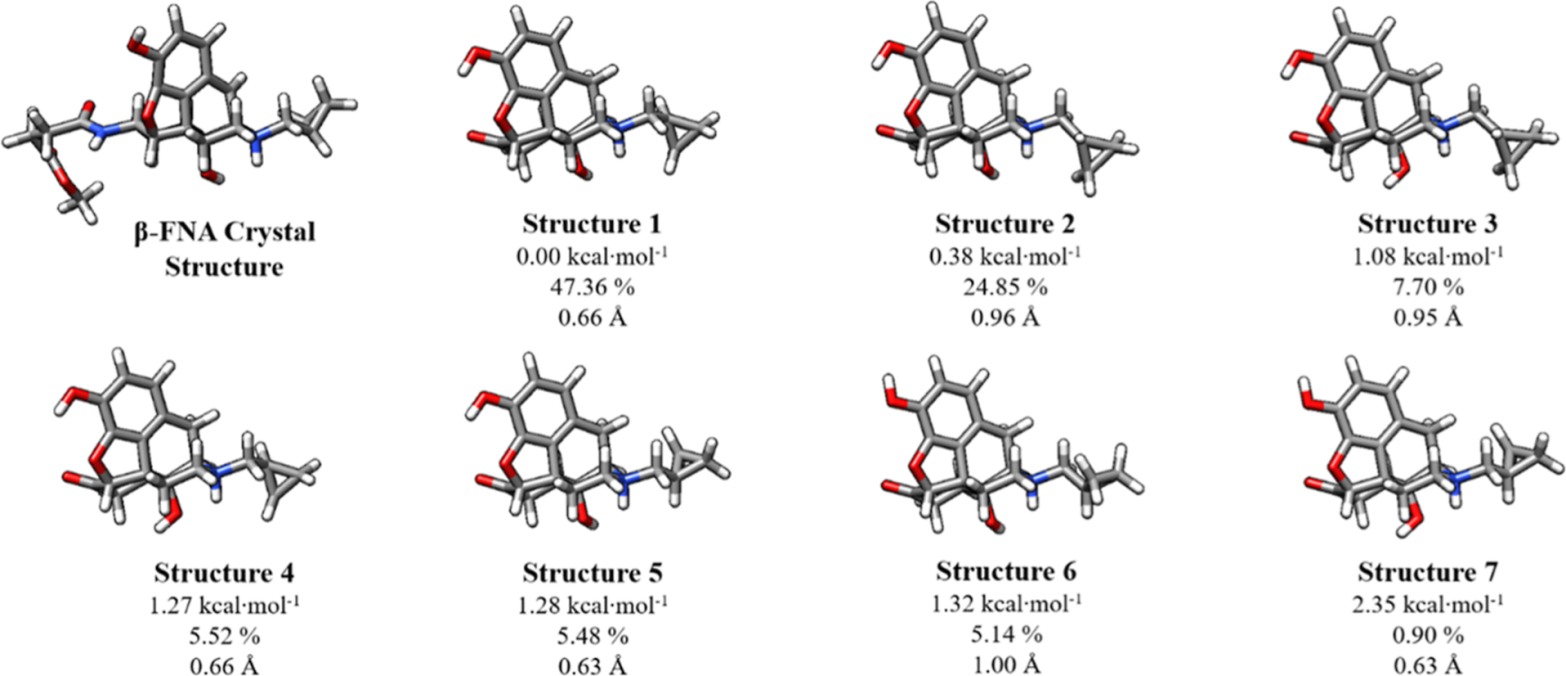
Lowest energy conformations of naltrexone at 310.15 K in aqueous solution generated at the ωB97X-D/6-31++G**/SMD level of theory. Structures are ordered in terms of Gibbs free energy (Δ*G*°) relative to the local minimum energy structure computed at the DLPNO-CCSD(T)/CBS/SMD//ωB97X-D/6-31++G**/SMD level of theory. Relative Gibbs free energies are shown in kcal·mol^–1^. Reported below each Δ*G*° are the percent abundance of each structure in aqueous solution at 310.15 K calculated using the Boltzmann equation. The RMSD of the heavy atoms for each structure in comparison to the conserved atoms of the crystallized β-FNA structure from the mMOR (PDB ID: 4DKL),^20^ which is included as a reference structure.

Naltrexone was used instead of β-FNA because the methyl-fumaramide group of β-FNA forms a covalent bond with the MOR, while naltrexone is truncated with a ketone. β-FNA binds irreversibly while naltrexone binds reversibly, so naltrexone is used as a mimic of the β-FNA antagonist. Naltrexone is similar to BU72 but is slightly more flexible because it has more rotatable bonds as shown in Figure 10. All structures are basically the same with RMSDs with respect to the lowest energy structure ranging from 0.63 to 1.00 Å, with only small changes in rotation about the OH and cyclopropyl groups. Boltzmann calculations reveal that all structures should be populated at physiological temperature and could easily change conformations. The Δ*G*° ranges from 0.38 kcal·mol^–1^ for structure 2 to 2.35 kcal·mol^–1^ for structure 7. We note that if structure 1 were to bind to MOR, then the equilibrium of the solution structures will shift, and the other conformers will shift to structure 1.

### Validation of the Glide Docking Program

**Figure 11:**
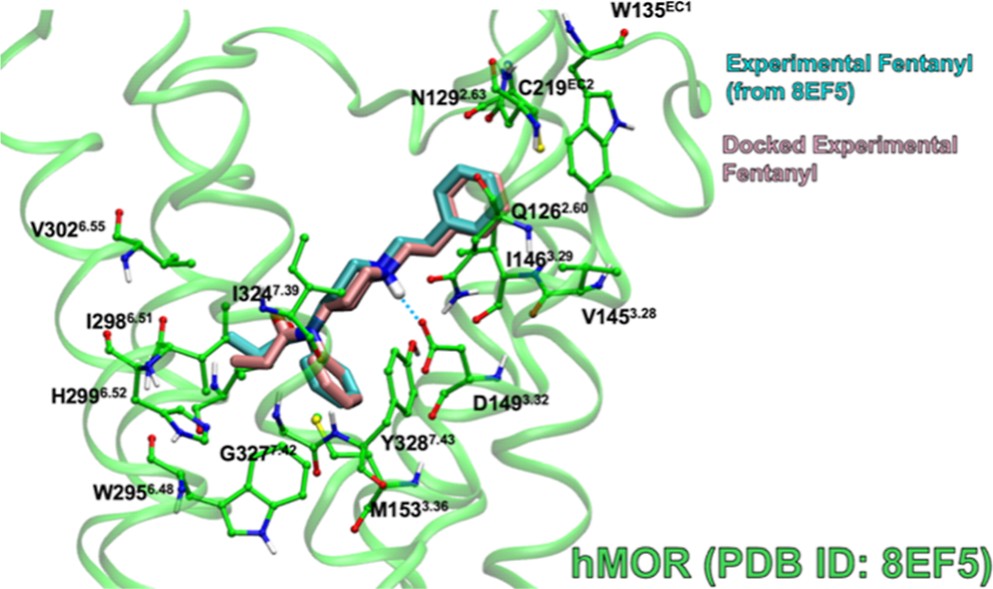
Best Glide docking-score complex of experimental fentanyl (pink, tube) rigidly docked to hMOR (green carbons, ball-and-stick side chains) versus the experimentally determined fentanyl (cyan, tube) (PDB ID: 8EF5).^10^ Binding site residues within 3 Å of the ligand are shown. Blue dashed lines indicate hydrogen bonds.

First, we verified that the Glide docking program can reproduce experimental results. The Glide program has been used successfully in a previous study^127^ to show how known experimental antagonists bind to the kappa opioid receptor. There was a positive correlation between the calculated docking score and the experimental binding affinities as evidence by a coefficient of determination of 0.92 out of a possible 1.0. While Glide’s docking score is not intended to be an accurate calculation of binding energies, the docking score does correlate with experimental binding affinities, so the docking scores are a reliable indicator of how strongly the receptor and ligand interact. Figure 11 shows the predicted binding modes of docking the experimental fentanyl structure to hMOR (PDB ID: 8EF5).^10^ The complex with the best docking score is in good agreement with the experimental poses. When the experimental fentanyl was docked rigidly, with ligand atoms frozen, the best docking score of the pose was −7.21 kcal·mol^–1^ with an RMSD of 0.59 Å with respect to the experimental ligand in the binding site. For comparison, merely minimizing the experimental fentanyl in the receptor produced a complex with a docking score of −6.96 kcal·mol^–1^ and an RMSD of 0.35 Å, which are comparable to the rigid docking results. This shows that the best docking score pose produced by Glide is valid because the program reproduced experimental results for fentanyl and hMOR.

**Figure 12:**
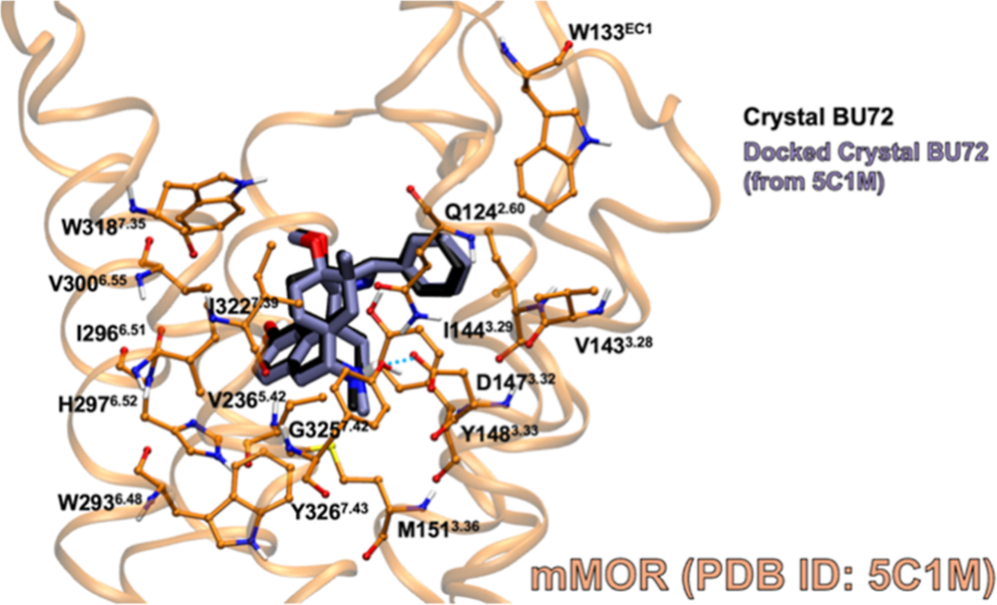
Best Glide docking-score complex of BU72 (purple, tube) in mMOR (PDB ID: 5C1M) without the N-terminus (orange, ball-and-stick side chains) versus BU72 (black, tube) experimentally crystallized with the complete mMOR (PDB ID: 5C1M).^8^ Binding site residues within 3 Å of the ligand are shown. Blue dashed lines indicate hydrogen bonds.

Glide docking was further validated by docking the crystallized BU72 ligand to mMOR (PDB ID: 5C1M)^8^ with the N-terminus removed. The N-terminus is absent in other models of MOR (PDB ID: 8EF5 and 4DKL), and since the N-terminus is in the BU72 binding site, the N-terminus residues interact with the bound ligand. Furthermore, there is controversy regarding how the N-terminus interacts with the BU72 ligand. Recently, Munro proposed that an oxygen atom forms a bridge between the nitrogen atoms in the ligand and the side-chain of His54,^30^ but the identity of this atom remains unknown. This interaction cannot be reproduced by our current docking methods. To standardize the binding site across all three receptor models and remove bias from the calculated docking score, the N-terminus was removed. The best docking score of BU72 to mMOR was −7.01 kcal·mol^–1^ (Figure 12) with an RMSD of 0.36 Å compared to the crystallized ligand. The RMSD and docking scores of these complexes are comparable to that of fentanyl to hMOR (−7.21 kcal·mol^–1^ and 0.59 Å, respectively), which further validates the Glide docking program and shows that the N-terminus is not necessary for accurately predicting the binding site of BU72.

### mMOR-Nanobody and hMOR-G_i_ Docking Comparison

**Figure 13:**
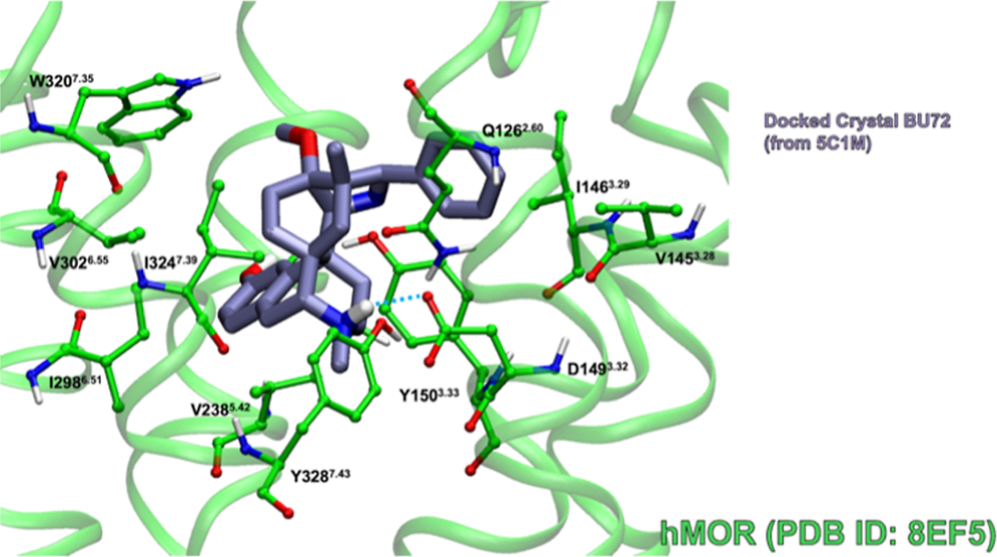
Best Glide docking-score complex of BU72 (purple, tube) to the hMOR (PDB ID: 8EF5)^10^ (green, ball-and-stick side chains). Binding site residues within 3 Å of the ligand are shown. Blue dashed lines indicate hydrogen bonds.

As stated above, the experimental mMOR-nanobody and hMOR-G_i_ bound structures differ given the type of intracellular protein that is bound to the intracellular end of the receptor. Here, we determine if these small structural changes have any impact on the binding of known agonists fentanyl and BU72. Figure 13 shows the complex of rigidly docked crystallized BU72 to experimental hMOR with the best-docking score of −6.09 kcal·mol^–1^. This docking score is less favorable than the corresponding value of BU72 docked to crystallized mMOR by only 1.03 kcal·mol^–1^. However, the Glide program did not produce any poses by docking experimental fentanyl to crystallized mMOR without the N-terminus. As mentioned above, the mMOR and hMOR experimental structures are similar, but even small structural differences impact ligand binding.

### Docking Predicted Fentanyl to hMOR (8EF5)

**Figure 14:**
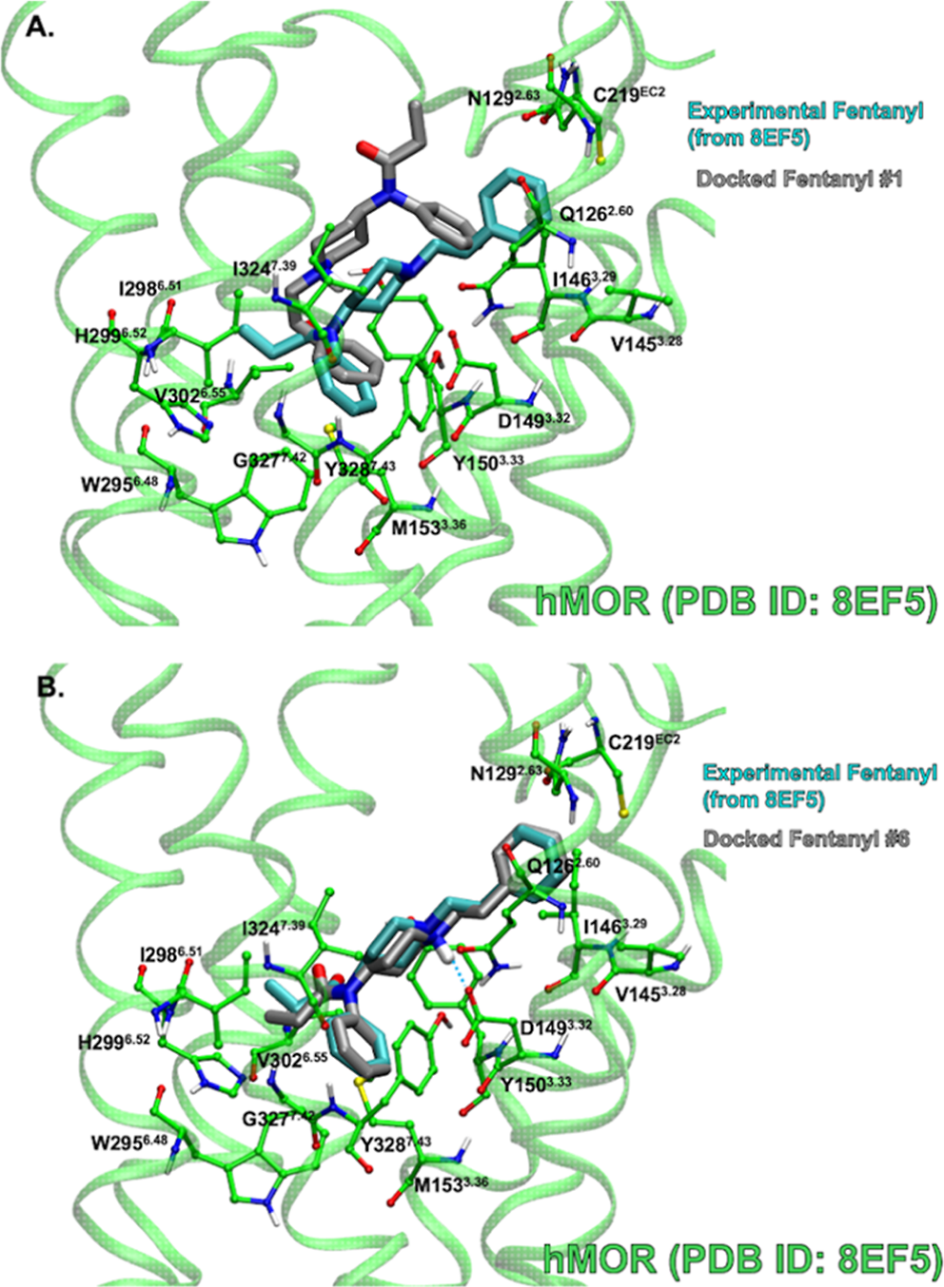
Experimental fentanyl (cyan, tube) versus best Glide docking-score complexes of the (A) lowest-energy fentanyl structure (gray, tube) and (B) sixth lowest-energy fentanyl (gray, tube) with hMOR (green, ball and stick side chains) (PDB ID: 8EF5). Binding site residues within 3 Å of the ligand are shown. Blue dashed lines indicate hydrogen bonds.

The lowest energy fentanyl structure in solution from the DLPNO-CCSD(T) calculations does not resemble the experimental fentanyl in complex with the hMOR. To determine if the low-energy solution structure binds favorably to hMOR, nine computationally predicted fentanyl structures were rigidly docked to experimental hMOR, and the best docking scores for each complex are shown in Table 1. The complex with the lowest-energy structure (Fentanyl 1 in Figure 8) had a score that was substantially more unfavorable by 4 kcal·mol^–1^ than that of the complex with the docked experimental ligand. A comparison of the two binding poses is shown in Figure 14A. The two ligand conformations found two different binding sites. Thus, the low-energy solution structure did not create a favorable or low-docking score complex with hMOR.

**Table 1:** Comparison of Docking Scores of Experimental Fentanyl and Quantum Mechanically Predicted Solution Fentanyl Structures Docked to hMOR (PDB ID: 8EF5)

protein	ligand	docking mode	docking score (kcal·mol^–1^)	RMSD with respect to the experimental ligand (Å)
8EF5	experimental fentanyl	rigid	–7.21	0.00
8EF5	fentanyl 1	rigid	–3.24	2.58
8EF5	fentanyl 2	rigid	–3.33	1.56
8EF5	fentanyl 3	rigid	–6.06	1.10
8EF5	fentanyl 4	rigid	–4.66	2.59
8EF5	fentanyl 5	rigid	–2.68	1.42
8EF5	fentanyl 6	rigid	–7.18	0.52
8EF5	fentanyl 7	rigid	–4.79	2.08
8EF5	fentanyl 8	rigid	–5.39	2.06
8EF5	fentanyl 9	rigid	–5.07	2.40

The computationally predicted fentanyl structure that produced the most favorable docking score was the sixth-lowest energy fentanyl structure in Figure 8. A visual comparison of this docked complex with the experimental complex shows good agreement between the ligand positioning in the binding site (Figure 14B) with an RMSD of 0.52 Å. Thus, the low-energy structures of fentanyl in solution do not necessarily correspond to favorable docking score complexes. Fentanyl has to undergo a conformational change to match the most favorable binding pose.

### Docking Predicted BU72 to mMOR (PDB ID: 5C1M)

For comparison, BU72, a morphinan agonist, was also quantum mechanically predicted and docked to mMOR for comparison with the docking of the crystallized ligand. Fewer conformations of BU72 than fentanyl were identified by quantum mechanical methods as shown in Figure 9, which is rationalized by the structure of the ligands. Fentanyl has several rotatable bonds whereas BU72 has multiple joined rings, so BU72 is more rigid and has less conformational flexibility than fentanyl. Unlike fentanyl, the low-energy solution structure of BU72 had a favorable docking score with crystallized mMOR (−6.49 kcal·mol^–1^) that is comparable to that of the docking of the crystallized BU72 (−7.01 kcal·mol^–1^) as shown in Table 2. All of the BU72 complexes have RMSDs of 0.58 Å or lower, so all of the docked ligand conformations are similar.

**Table 2:** Comparison of Docking Scores of Crystallized BU72 and Quantum Mechanically Predicted BU72 Structures from Figure 9 Docked to mMOR (PDB ID: 5C1M) without the N-Terminus

protein	ligand	docking mode	docking score (kcal·mol^–1^)	RMSD with respect to the crystallized ligand (Å)
5C1M (no N-term)	crystal BU72	rigid	–7.01	0.00
5C1M (no N-term)	BU72 1	rigid	–6.49	0.27
5C1M (no N-term)	BU72 2	rigid	–6.47	0.58
5C1M (no N-term)	BU72 3	rigid	–6.43	0.53

### Docking Predicted Naltrexone to mMOR (PDB ID: 4DKL)

Quantum mechanical methods were used to predict several low-energy structures of naltrexone, a morphinan antagonist as seen in Figure 10. Naltrexone is a derivative of the crystallized β-FNA with mMOR. Since β-FNA forms a covalent bond with K233^5.39^ in mMOR, the Glide docking program cannot successfully reproduce docking complexes that agree with the experimental crystal structure (PDB ID: 4DKL(20)) because the program cannot predict covalent bond formation. However, given the conformational similarity of naltrexone and β-FNA, it is anticipated that naltrexone would match the binding site of β-FNA. Table 3 shows the predicted docking scores of seven naltrexone conformations from Figure 10 to crystallized mMOR. The naltrexone conformation that was a modified version of the crystallized β-FNA had the most favorable docking score of −8.49 kcal·mol^–1^. The lowest-energy structure of naltrexone in solution had the most second-most favorable docking score to mMOR (−7.79 kcal·mol^–1^). However, the fifth-lowest energy structure of naltrexone had a comparable docking score (−7.77 kcal·mol^–1^), which is essentially an identical docking score given the approximations used in Glide program calculations. This is consistent with the trend observed in fentanyl in that the higher energy ligand structures in solution can create favorable complexes with the receptors. On the other hand, all of the conformations in solution are structurally similar to the derivative of the crystal structure with RMSDs ranging from 0.47 to 0.89 Å with respect to the crystal-derived structure in Figure 10. In addition, the Boltzmann populations indicate that all structures are available in solution, so that 100% of naltrexone low-energy structures in solution should bind to the MOR.

**Table 3:** Comparison of Docking Scores of Quantum Mechanically Predicted Naltrexone Structures Docked to mMOR (PDB ID: 4DKL)

protein	ligand	docking mode	docking score (kcal·mol^–1^)	RMSD with respect to the crystallized ligand(Å)
4DKL	crystal naltrexone	rigid	–8.49	0.00
4DKL	naltrexone 1	rigid	–7.79	0.54
4DKL	naltrexone 2	rigid	–6.26	0.86
4DKL	naltrexone 3	rigid	–5.09	0.85
4DKL	naltrexone 4	rigid	–4.16	0.53
4DKL	naltrexone 5	rigid	–7.77	0.47
4DKL	naltrexone 6	rigid	–5.81	0.89
4DKL	naltrexone 7	rigid	–4.89	0.47

**Figure 15:**
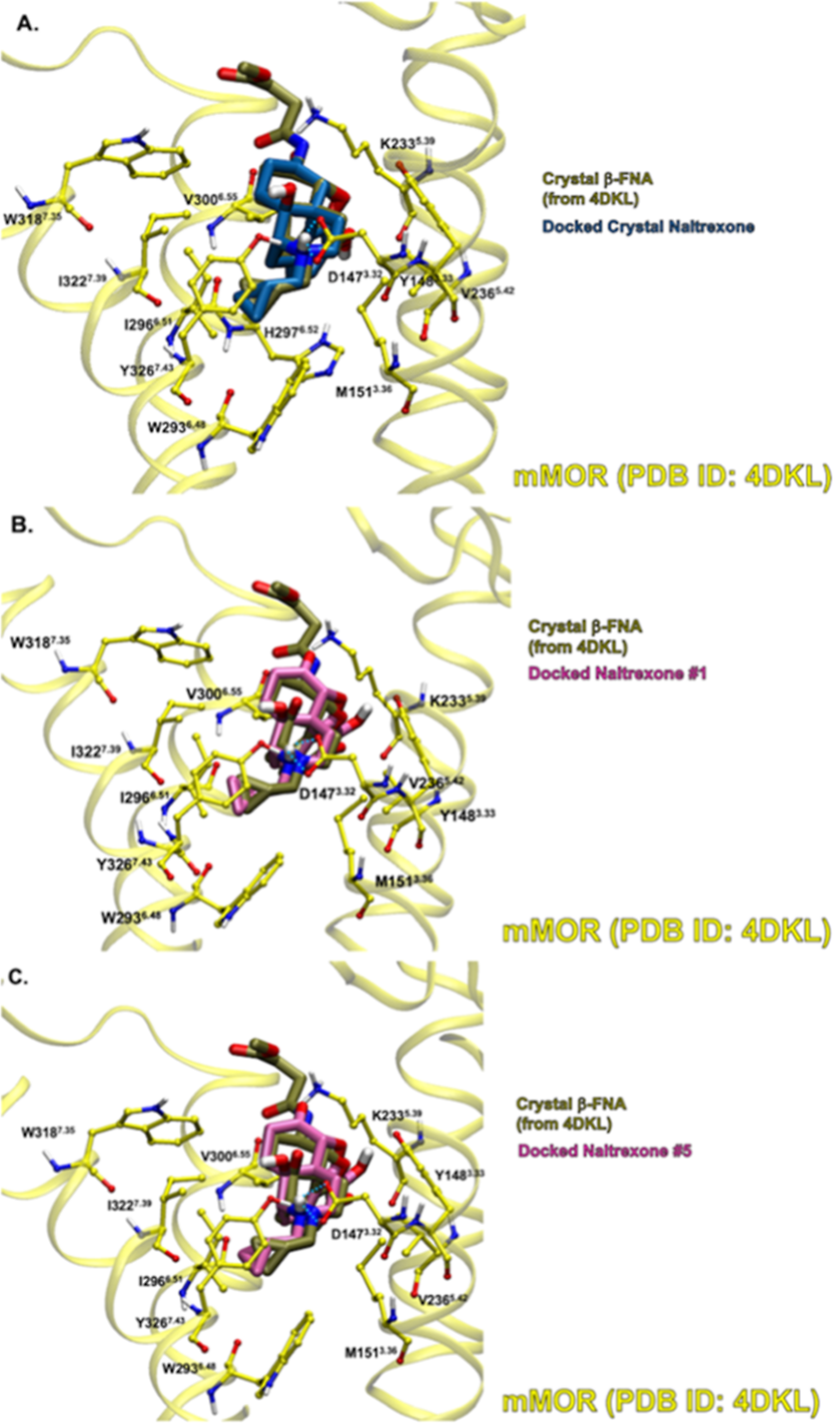
Crystallized β-FNA (brown, tube) versus best Glide docking-score complexes of (A) naltrexone derived from crystallized β-FNA (blue, tube), (B) lowest-energy naltrexone (magenta, tube), and (C) fifth lowest-energy naltrexone (magenta, tube) with mMOR (yellow, ball and stick side chains) (PDB ID: 4DKL). Binding site residues within 3 Å of the ligand are shown. Blue dashed lines indicate hydrogen bonds.

As described above, it was hypothesized that naltrexone would have a binding site in mMOR that is similar to that of β-FNA. Figure 15A shows the predicted binding site of the naltrexone structure derived from the crystallized β-FNA in comparison to the β-FNA in mMOR. There is good agreement between conserved portions of the ligands. Figure 15B,C show the predicted binding sites of the lowest-energy naltrexone and the fifth-lowest energy naltrexone in mMOR, respectively, in comparison with the crystallized β-FNA. Both quantum-mechanically predicted ligand conformations have docked poses that match β-FNA as anticipated. The RMSDs of Naltrexone 1 and 5 with respect to the β-FNA-derived naltrexone are 0.54 and 0.47 Å, respectively. Thus, the poses with favorable docking scores agree with the β-FNA poses.

## Conclusions

Experimental receptor–ligand complexes determined by X-ray crystallography and cryogenic electron microscopy provide a great deal of structural and functional information. Despite the recent increase in the number of crystallized GPCRs, the experimental data on opioids binding to hMORs remain limited. For example, at the time of writing this article, there is only one experimentally determined structure of hMOR bound to fentanyl. Our study uses quantum mechanical and docking methods to demonstrate that there is structural diversity in receptors and ligands and that there are multiple possible receptor–ligand complexes. It is well-known that GPCRs, such as MORs, can bind to a variety of intracellular proteins, so it stands to reason that these different intracellular proteins stabilize different receptor conformations. Principal component analysis shows that there are significant differences between active and inactive structures and the G_i_ versus nanobody-bound structures, which agrees with Munro’s reanalysis of the crystal structure of nanobody-bound mMOR.^30^ This calls into question the appropriateness of using nanobody-bound MORs as models for the active conformation. Small changes in the receptor structure impact the binding site of the ligands, and therefore, the binding mode of the ligands, so an accurate receptor structure is vital for modeling and drug design.

We have completed a thorough conformational analysis to determine the low energy structures of the agonists fentanyl and BU72 and the antagonist naltrexone in solution. The lowest energy fentanyl structure differs from the experimental structure bound to the hMOR-G_i_ complex, and p*K*_a_ calculations reveal that fentanyl is protonated in aqueous solution. This ligand must undergo a conformational change to obtain a higher energy state to bind favorably to the receptor. On the other hand, the lowest-energy solution structures of the morphinan agonist BU72 and the antagonist naltrexone do closely resemble the crystal structures bound to the respective mMORs. Our computational studies have determined the respective energetic barriers that the ligand must overcome to bind to the receptor. Docking results show that the most favorable scoring complexes have higher energy ligand conformations. However, docking results also demonstrate that it is possible for ligand conformations that differ from the experimental ligand pose to bind to the receptor. Here, we determined the relative population of each ligand in aqueous solution at 310.15 K and show that a variety of ligand poses can bind to the receptor. The Boltzmann distribution shows that nine varied fentanyl conformations but only three similar BU72 and seven similar naltrexone conformations are accessible at body temperature. The more flexible fentanyl molecule docks with multiple poses, which may explain why fentanyl induces multiple intracellular protein binding and multiple signaling pathways, resulting in different physiological effects including pain relief and adverse side effects. More research on the conformations and binding modes of flexible opioids should be pursued to further elucidate the structure–activity relationship in hMOR.

## Supporting Information Available

DLPNO-CCSD(T)/cc-pVnZ/SMD (n = D,T,Q) electronic energies and extrapolation to the complete basis set (CBS) limit; optimized geometries of all ligand structures within 3 kcal/mol of the Δ*G*° minimum computed with ωB97X-D/6-31++G**/SMD model chemistry; derivation of the CBS extrapolation formula; the coordinates of the docked complexes and the extracted docked ligands (ZIP)

## References

[ref1] The Council on Economic Advisers. The Underestimated Cost of the Opioid Crisis, 2017. https://www.whitehouse.gov (accessed 2024-10-22).

[ref2] Reidhead M.; Billings S. The Economic Cost of the Opioid Crisis in the U.S. A State-by-State Comparison, 2019. https://www.mhanet.com/mhaimages/Policy_Briefs/PolicyBrief_Economic_Cost_ofthe_Opioid_Crisis_inthe_U.S._0419.pdf (accessed 2024-10-22).

[ref3] Rikard S. M.; Strahan A. E.; Schmit K. M.; Guy G. P. Chronic Pain Among Adults–United States, 2019–2021. MMWR Morb Mortal Wkly Rep 2023, 72, 379–385. 10.15585/mmwr.mm7215a1. 37053114 PMC10121254

[ref4] Understanding the Opioid Overdose Epidemic. https://www.cdc.gov/opioids/basics/epidemic.html (accessed 2024-02-15).

[ref5] Hedayati-Moghadam M.; Moezi S. A.; Kazemi T.; Sami A.; Akram M.; Zainab R.; Khazdair M. R. The effects of Papaver somniferum (Opium poppy) on health, its controversies and consensus evidence. Toxin Rev. 2022, 41, 1030–1043. 10.1080/15569543.2021.1958232.

[ref6] Drug Fact Sheet: Synthetic Opioids. https://www.dea.gov/sites/default/files/2020-06/Synthetic%20Opioids-2020.pdf (accessed 2024-02-15).

[ref7] Hedegaard H.; Miniño A. M.; Spencer M. R.; Warner M. Drug Overdose Deaths in the United States, 1999–2020: Hyattsville, MD, 2021. 34978529

[ref8] Huang W.; Manglik A.; Venkatakrishnan A. J.; Laeremans T.; Feinberg E. N.; Sanborn A. L.; Kato H. E.; Livingston K. E.; Thorsen T. S.; Kling R. C.; et al. Structural insights into μ-opioid receptor activation. Nature 2015, 524, 315–321. 10.1038/nature14886. 26245379 PMC4639397

[ref9] Johnston J. M.; Filizola M. Beyond Standard Molecular Dynamics: Investigating the Molecular Mechanisms of G Protein-Coupled Receptors with Enhanced Molecular Dynamics Methods. In G Protein-Coupled Receptors–Modeling and Simulation; Filizola M., Ed.; Springer Netherlands: Dordrecht, 2014; pp 95–125. 10.1007/978-94-007-7423-0_6 PMC4074508 24158803

[ref10] Zhuang Y.; Wang Y.; He B.; He X.; Zhou X. E.; Guo S.; Rao Q.; Yang J.; Liu J.; Zhou Q.; et al. Molecular recognition of morphine and fentanyl by the human μ-opioid receptor. Cell 2022, 185, 4361 10.1016/j.cell.2022.09.041. 36368306

[ref11] Wang H.; Hetzer F.; Huang W.; Qu Q.; Meyerowitz J.; Kaindl J.; Hübner H.; Skiniotis G.; Kobilka B. K.; Gmeiner P. Structure-Based Evolution of G Protein-Biased μ-Opioid Receptor Agonists. Angew. Chem., Int. Ed. 2022, 61, e202200269 10.1002/anie.202200269. PMC9322534 35385593

[ref12] Shukla A. K.; Dwivedi-Agnihotri H. Structure and function of β-arrestins, their emerging role in breast cancer, and potential opportunities for therapeutic manipulation. Adv. Cancer Res. 2020, 145, 139–156. 10.1016/bs.acr.2020.01.001. 32089163 PMC7115872

[ref13] Ballesteros J. A.; Weinstein H. [19] Integrated methods for the construction of three-dimensional models and computational probing of structure-function relations in G protein-coupled receptors. In Methods in Neurosciences; Sealfon S. C., Ed.; Academic Press, 1995; Vol. 25, pp 366–428. 10.1016/s1043-9471(05)80049-7.

[ref14] Herman T. F.; Cascella M.; Muzio M. R. Mu Receptors. In StatPearls; StatPearls Publishing: Treasure Island, FL, 2024. 31855381

[ref15] Connor M.; Christie M. J. Opioid receptor signalling mechanisms. Clin. Exp. Pharmacol. Physiol. 1999, 26, 493–499. 10.1046/j.1440-1681.1999.03049.x. 10405772

[ref16] Raehal K. M.; Walker J. K.; Bohn L. M. Morphine Side Effects in β-Arrestin 2 Knockout Mice. J. Pharmacol. Exp. Ther. 2005, 314, 1195–1201. 10.1124/jpet.105.087254. 15917400

[ref17] Bohn L. M.; Lefkowitz R. J.; Caron M. G. Differential Mechanisms of Morphine Antinociceptive Tolerance Revealed in βArrestin-2 Knock-Out Mice. J. Neurosci. 2002, 22, 10494–10500. 10.1523/JNEUROSCI.22-23-10494.2002. 12451149 PMC6758751

[ref18] Bohn L. M.; Lefkowitz R. J.; Gainetdinov R. R.; Peppel K.; Caron M. G.; Lin F. T. Enhanced Morphine Analgesia in Mice Lacking β-Arrestin 2. Science 1999, 286, 2495–2498. 10.1126/science.286.5449.2495. 10617462

[ref19] Bayron J. A.; Deveau A. M.; Stubbs J. M. Conformational Analysis of 6α- and 6β-Naltrexol and Derivatives and Relationship to Opioid Receptor Affinity. J. Chem. Inf. Model. 2012, 52, 391–395. 10.1021/ci200405u. 22263545

[ref20] Manglik A.; Kruse A. C.; Kobilka T. S.; Thian F. S.; Mathiesen J. M.; Sunahara R. K.; Pardo L.; Weis W. I.; Kobilka B. K.; Granier S. Crystal structure of the μ-opioid receptor bound to a morphinan antagonist. Nature 2012, 485, 321–326. 10.1038/nature10954. 22437502 PMC3523197

[ref21] Kaserer T.; Lantero A.; Schmidhammer H.; Spetea M.; Schuster D. μ Opioid receptor: novel antagonists and structural modeling. Sci. Rep. 2016, 6, 21548 10.1038/srep21548. 26888328 PMC4757823

[ref22] Brederson J. D.; Kym P. R.; Szallasi A. Targeting TRP channels for pain relief. Eur. J. Pharmacol. 2013, 716, 61–76. 10.1016/j.ejphar.2013.03.003. 23500195

[ref23] Koehl A.; Hu H.; Maeda S.; Zhang Y.; Qu Q.; Paggi J. M.; Latorraca N. R.; Hilger D.; Dawson R.; Matile H.; et al. Structure of the μ-opioid receptor–Gi protein complex. Nature 2018, 558, 547–552. 10.1038/s41586-018-0219-7. 29899455 PMC6317904

[ref24] Davis R. L.; Das S.; Thomas Curtis J.; Stevens C. W. The opioid antagonist, β-funaltrexamine, inhibits NF-κB signaling and chemokine expression in human astrocytes and in mice. Eur. J. Pharmacol. 2015, 762, 193–201. 10.1016/j.ejphar.2015.05.040. 26007645 PMC4543532

[ref25] Modesto-Lowe V.; Van Kirk J. Clinical uses of naltrexone: A review of the evidence. Exp. Clin. Psychopharmacol. 2002, 10, 213–227. 10.1037/1064-1297.10.3.213. 12233982

[ref26] Oslin D. W.; Leong S. H.; Lynch K. G.; Berrettini W.; O’Brien C. P.; Gordon A. J.; Rukstalis M. Naltrexone vs Placebo for the Treatment of Alcohol Dependence: A Randomized Clinical Trial. JAMA Psychiatry 2015, 72, 430–437. 10.1001/jamapsychiatry.2014.3053. 25760804

[ref27] Toljan K.; Vrooman B. Low-Dose Naltrexone (LDN)—Review of Therapeutic Utilization. Med. Sci. 2018, 6, 82 10.3390/medsci6040082. PMC6313374 30248938

[ref28] Sounier R.; Mas C.; Steyaert J.; Laeremans T.; Manglik A.; Huang W.; Kobilka B. K.; Déméné H.; Granier S. Propagation of conformational changes during μ-opioid receptor activation. Nature 2015, 524, 375–378. 10.1038/nature14680. 26245377 PMC4820006

[ref29] Neilan C. L.; Husbands S. M.; Breeden S.; Ko M. H.; Aceto M. D.; Lewis J. W.; Woods J. H.; Traynor J. R. Characterization of the complex morphinan derivative BU72 as a high efficacy, long-lasting mu-opioid receptor agonist. Eur. J. Pharmacol. 2004, 499, 107–116. 10.1016/j.ejphar.2004.07.097. 15363957

[ref30] Munro T. A. Reanalysis of a μ opioid receptor crystal structure reveals a covalent adduct with BU72. BMC Biology 2023, 21, 213 10.1186/s12915-023-01689-w. 37817141 PMC10566028

[ref31] Munro T. A. Revised (β-phenyl) stereochemistry of ultrapotent μ opioid BU72. bioRxiv 2020, 10.1101/2020.04.01.020883v1.

[ref32] Schmid C. L.; Kennedy N. M.; Ross N. C.; Lovell K. M.; Yue Z.; Morgenweck J.; Cameron M. D.; Bannister T. D.; Bohn L. M. Bias Factor and Therapeutic Window Correlate to Predict Safer Opioid Analgesics. Cell 2017, 171, 1165–1175. 10.1016/j.cell.2017.10.035. 29149605 PMC5731250

[ref33] Podlewska S.; Bugno R.; Kudla L.; Bojarski A. J.; Przewlocki R. Molecular Modeling of μ Opioid Receptor Ligands with Various Functional Properties: PZM21, SR-17018, Morphine, and Fentanyl—Simulated Interaction Patterns Confronted with Experimental Data. Molecules 2020, 25, 4636 10.3390/molecules25204636. 33053718 PMC7594085

[ref34] Lipiński P. F. J.; Jarończyk M.; Dobrowolski J. C.; Sadlej J. Molecular dynamics of fentanyl bound to μ-opioid receptor. J Mol Model 2019, 25, 144 10.1007/s00894-019-3999-2. 31053968

[ref35] de Waal P. W.; Shi J.; You E.; Wang X.; Melcher K.; Jiang Y.; Xu H. E.; Dickson B. M. Molecular mechanisms of fentanyl mediated β-arrestin biased signaling. PLoS Comput. Biol. 2020, 16, e1007394 10.1371/journal.pcbi.1007394. 32275713 PMC7176292

[ref36] Xie B.-G.; Goldberg A.; Shi L. A comprehensive evaluation of the potential binding poses of fentanyl and its analogs at the μ-opioid receptor. Comput. Struct. Biotechnol. J. 2022, 20, 2309–2321. 10.1016/j.csbj.2022.05.013. 35615021 PMC9123087

[ref37] Vo Q. N.; Mahinthichaichan P.; Shen J.; Ellis C. R. How μ-opioid receptor recognizes fentanyl. Nat. Commun. 2021, 12, 984 10.1038/s41467-021-21262-9. 33579956 PMC7881245

[ref38] Marenich A. V.; Cramer C. J.; Truhlar D. G. Universal Solvation Model Based on Solute Electron Density and on a Continuum Model of the Solvent Defined by the Bulk Dielectric Constant and Atomic Surface Tensions. J. Phys. Chem. B. 2009, 113, 6378–6396. 10.1021/jp810292n. 19366259

[ref39] Pracht P.; Bohle F.; Grimme S. Automated exploration of the low-energy chemical space with fast quantum chemical methods. Phys. Chem. Chem. Phys. 2020, 22, 7169–7192. 10.1039/C9CP06869D. 32073075

[ref40] Grimme S. Exploration of Chemical Compound, Conformer, and Reaction Space with Meta-Dynamics Simulations Based on Tight-Binding Quantum Chemical Calculations. J. Chem. Theory Comput. 2019, 15, 2847–2862. 10.1021/acs.jctc.9b00143. 30943025

[ref41] Bannwarth C.; Ehlert S.; Grimme S. GFN2-xTB-An Accurate and Broadly Parametrized Self-Consistent Tight-Binding Quantum Chemical Method with Multipole Electrostatics and Density-Dependent Dispersion Contributions. J. Chem. Theory Comput. 2019, 15, 1652–1671. 10.1021/acs.jctc.8b01176. 30741547

[ref42] Chai J. D.; Head-Gordon M. Long-range corrected hybrid density functionals with damped atom-atom dispersion corrections. Phys. Chem. Chem. Phys. 2008, 10, 6615–6620. 10.1039/b810189b. 18989472

[ref43] Chai J. D.; Head-Gordon M. Systematic optimization of long-range corrected hybrid density functionals. J. Chem. Phys. 2008, 128, 084106 10.1063/1.2834918. 18315032

[ref44] Odbadrakh T. T.; Gale A. G.; Ball B. T.; Temelso B.; Shields G. C. Computation of atmospheric concentrations of molecular clusters from ab initio thermochemistry. J. Visualized Exp. 2020, 158, e60964 10.3791/60964. 32338653

[ref45] Ball B. T.; Vanovac S.; Odbadrakh T. T.; Shields G. C. Monomers of glycine and serine have a limited ability to hydrate in the atmosphere. J. Phys. Chem. A. 2021, 125, 8454–8467. 10.1021/acs.jpca.1c05466. 34529444

[ref46] Ditchfield R.; Hehre W. J.; Pople J. A. Self-consistent molecular-orbital methods. IX. An extended Gaussian-type basis for molecular-orbital studies of organic molecules. J. Chem. Phys. 1971, 54, 724–728. 10.1063/1.1674902.

[ref47] Hehre W. J.; Ditchfield R.; Pople J. A. Self-consistent molecularorbital methods. XII. Further extensions of Gaussian-type basis sets for use in molecular orbital studies of organic molecules. J. Chem. Phys. 1972, 56, 2257–2261. 10.1063/1.1677527.

[ref48] Hariharan P. C.; Pople J. A. The influence of polarization functions on molecular orbital hydrogenation energies. Theor. Chim. Acta 1973, 28, 213–222. 10.1007/BF00533485.

[ref49] Francl M. M.; Pietro W. J.; Hehre W. J.; Binkley J. S.; Gordon M. S.; DeFrees D. J.; Pople J. A. Self-consistent molecular orbital methods. XXIII. A polarization-type basis set for second-row elements. J. Chem. Phys. 1982, 77, 3654–3665. 10.1063/1.444267.

[ref50] Frisch M. J.; Pople J. A.; Binkley J. S. Self-consistent molecular orbital methods 25. Supplementary functions for Gaussian basis sets. J. Chem. Phys. 1984, 80, 3265–3269. 10.1063/1.447079.

[ref51] Frisch M. J.; Trucks G. W.; Schlegel H. B.; Scuseria G. E.; Robb M. A.; Cheeseman J. R.; Scalmani G.; Barone V.; Petersson G. A.; Nakatsuji H. L. X.; Gaussian 16. Revision B.01; Gaussian Inc: Wallingford, CT, 2016.

[ref52] Irikura K. K. Thermo.PL; NIST, 2002.

[ref53] Neese F. Prediction of molecular properties and molecular spectroscopy with density functional theory: From fundamental theory to exchange-coupling. Coord. Chem. Rev. 2009, 253, 526–563. 10.1016/j.ccr.2008.05.014.

[ref54] Neese F.; Hansen A.; Liakos D. G. Efficient and accurate approximations to the local coupled cluster singles doubles method using a truncated pair natural orbital basis. J. Chem. Phys. 2009, 131 (6), 064103 10.1063/1.3173827. 19691374

[ref55] Neese F.; Hansen A.; Wennmohs F.; Grimme S. Accurate Theoretical Chemistry with Coupled Pair Models. Acc. Chem. Res. 2009, 42, 641–648. 10.1021/ar800241t. 19296607

[ref56] Neese F.; Wennmohs F.; Hansen A. Efficient and accurate local approximations to coupled-electron pair approaches: An attempt to revive the pair natural orbital method. J. Chem. Phys. 2009, 130 (11), 114108 10.1063/1.3086717. 19317532

[ref57] Hansen A.; Liakos D. G.; Neese F. Efficient and accurate local single reference correlation methods for high-spin open-shell molecules using pair natural orbitals. J. Chem. Phys. 2011, 135, 214102 10.1063/1.3663855. 22149774

[ref58] Liakos D. G.; Neese F. Improved correlation energy extrapolation schemes based on local pair natural orbital methods. J. Phys. Chem. A 2012, 116, 4801–4816. 10.1021/jp302096v. 22489633

[ref59] Riplinger C.; Neese F. An efficient and near linear scaling pair natural orbital based local coupled cluster method. J. Chem. Phys. 2013, 138, 034106 10.1063/1.4773581. 23343267

[ref60] Riplinger C.; Sandhoefer B.; Hansen A.; Neese F. Natural triple excitations in local coupled cluster calculations with pair natural orbitals. J. Chem. Phys. 2013, 139, 134101 10.1063/1.4821834. 24116546

[ref61] Liakos D. G.; Neese F. Is it possible to obtain coupled cluster quality energies at near density functional theory cost? Domain-based local pair natural orbital coupled cluster vs modern density functional theory. J. Chem. Theory Comput. 2015, 11, 4054–4063. 10.1021/acs.jctc.5b00359. 26575901

[ref62] Liakos D. G.; Sparta M.; Kesharwani M. K.; Martin J. M.; Neese F. Exploring the accuracy limits of local pair natural orbital coupled-cluster theory. J. Chem. Theory Comput. 2015, 11, 1525–1539. 10.1021/ct501129s. 26889511

[ref63] Riplinger C.; Pinski P.; Becker U.; Valeev E. F.; Neese F. SparseMaps - A systematic infrastructure for reduced-scaling electronic structure methods. II. Linear scaling domain based pair natural orbital coupled cluster theory. J. Chem. Phys. 2016, 144, 024109 10.1063/1.4939030. 26772556

[ref64] Pavosevic F.; Peng C.; Pinski P.; Riplinger C.; Neese F.; Valeev E. F. SparseMaps - A systematic infrastructure for reduced-scaling electronic structure methods. V. Linear scaling explicitly correlated coupled-cluster method with pair natural orbitals. J. Chem. Phys. 2017, 146, 174108 10.1063/1.4979993. 28477585

[ref65] Sparta M.; Retegan M.; Pinski P.; Riplinger C.; Becker U.; Neese F. Multilevel approaches within the local pair natural orbital framework. J. Chem. Theory Comput. 2017, 13, 3198–3207. 10.1021/acs.jctc.7b00260. 28590754

[ref66] Guo Y.; Riplinger C.; Becker U.; Liakos D. G.; Minenkov Y.; Cavallo L.; Neese F. Communication: An improved linear scaling perturbative triples correction for the domain based local pair-natural orbital based singles and doubles coupled cluster method [DLPNO-CCSD(T)]. J. Chem. Phys. 2018, 148, 011101 10.1063/1.5011798. 29306283

[ref67] Kurfman L. A.; Odbadrakh T. T.; Shields G. C. Calculating Reliable Gibbs Free Energies for Formation of Gas-Phase Clusters that Are Critical for Atmospheric Chemistry: (H(2)SO(4))(3). J. Phys. Chem. A 2021, 125, 3169–3176. 10.1021/acs.jpca.1c00872. 33825467

[ref68] Elm J.; Ayoubi D.; Engsvang M.; Jensen A. B.; Knattrup Y.; Kubečka J.; Bready C. J.; Fowler V. R.; Harold S. E.; Longsworth O. M.; et al. Quantum chemical modeling of organic enhanced atmospheric nucleation: A critical review. WIREs Comput. Mol. Sci. 2023, 13, e1662 10.1002/wcms.1662.

[ref69] Neese F. Software update: The ORCA program system—Version 5.0. WIREs Comput. Mol. Sci. 2022, 12, e1606 10.1002/wcms.1606.

[ref70] Dunning T. H. Gaussian basis sets for use in correlated molecular calculations. I. The atoms boron through neon and hydrogen. J. Chem. Phys. 1989, 90, 1007–1023. 10.1063/1.456153.

[ref71] Kendall R. A.; Dunning T. H.; Harrison R. J. Electron affinities of the first-row atoms revisited. Systematic basis sets and wave functions. J. Chem. Phys. 1992, 96, 6796–6806. 10.1063/1.462569.

[ref72] Wilson A. K.; van Mourik T.; Dunning T. H. Gaussian basis sets for use in correlated molecular calculations. VI. Sextuple zeta correlation consistent basis sets for boron through neon. J. Mol. Struct. 1996, 388, 339–349. 10.1016/s0166-1280(96)04689-1.

[ref73] Helgaker T.; Klopper W.; Koch H.; Noga J. Basis-set convergence of correlated calculations on water. J. Chem. Phys. 1997, 106, 9639–9646. 10.1063/1.473863.

[ref74] Temelso B.; Mabey J. M.; Kubota T.; Appiah-Padi N.; Shields G. C. ArbAlign: A tool for optimal alignment of arbitrarily ordered isomers using the Kuhn-Munkres Algorithm. J. Chem. Theory Comput. 2017, 57, 1045–1054. 10.1021/acs.jcim.6b00546. 28398732

[ref75] Hanwell M. D.; Curtis D. E.; Lonie D. C.; Vandermeersch T.; Zurek E.; Hutchison G. R. Avogadro: an advanced semantic chemical editor, visualization, and analysis platform. J. Cheminf. 2012, 4, 17 10.1186/1758-2946-4-17. PMC3542060 22889332

[ref76] Pettersen E. F.; Goddard T. D.; Huang C. C.; Couch G. S.; Greenblatt D. M.; Meng E. C.; Ferrin T. E. UCSF Chimera-a visualization system for exploratory research and analysis. J. Comput. Chem. 2004, 25, 1605–1612. 10.1002/jcc.20084. 15264254

[ref77] Alongi K. S.; Shields G. C. Theoretical Calculations of Acid Dissociation Constants: A Review Article. In Annual Reports in Computational Chemistry, Vol 6; Wheeler R. A., Ed.; Elsevier Science Bv: Amsterdam, 2010; Vol. 6, pp 113–138. 10.1016/s1574-1400(10)06008-1.

[ref78] Shields G. C.; Seybold P. G., Computational Approaches for the Prediction of pK_a_ Values. Hardback, Ed.; CRC Press: Boca Raton, 2014.

[ref79] Seybold P. G.; Shields G. C. Computational estimation of pK(a) values. Wiley Interdiscip. Rev. Comput. Mol. Sci. 2015, 5, 290–297. 10.1002/wcms.1218.

[ref80] Liptak M. D.; Shields G. C. Experimentation with different thermodynamic cycles used for p*K*_a_ calculations on carboxylic acids using complete basis set and Gaussian-*n* models combined with CPCM continuum solvation methods. Int. J. Quantum Chem. 2001, 85, 727–741. 10.1002/qua.1703. 11472159

[ref81] Liptak M. D.; Shields G. C. Accurate pK(a) calculations for carboxylic acids using complete basis set and Gaussian-n models combined with CPCM continuum solvation methods. J. Am. Chem. Soc. 2001, 123, 7314–7319. 10.1021/ja010534f. 11472159

[ref82] Berman H. M.; Westbrook J.; Feng Z.; Gilliland G.; Bhat T. N.; Weissig H.; Shindyalov I. N.; Bourne P. E. The Protein Data Bank. Nucleic Acids Res. 2000, 28, 235–242. 10.1093/nar/28.1.235. 10592235 PMC102472

[ref83] Berman H.; Henrick K.; Nakamura H. Announcing the worldwide Protein Data Bank. Nat. Struct. Mol. Biol. 2003, 10, 980 10.1038/nsb1203-980. 14634627

[ref84] Humphrey W.; Dalke A.; Schulten K. VMD: visual molecular dynamics. J Mol Graph 1996, 14, 33–38. 10.1016/0263-7855(96)00018-5. 8744570

[ref85] Grant B. J.; Skjærven L.; Yao X.-Q. The Bio3D packages for structural bioinformatics. Protein Sci. 2021, 30, 20–30. 10.1002/pro.3923. 32734663 PMC7737766

[ref86] Scott C. E.; Kekenes-Huskey P. M. Molecular Basis of S100A1 Activation at Saturating and Subsaturating Calcium Concentrations. Biophys. J. 2016, 110, 1052–1063. 10.1016/j.bpj.2015.12.040. 26958883 PMC4788715

[ref87] Altschul S. F.; Madden T. L.; Schaffer A. A.; Zhang J.; Zhang Z.; Miller W.; Lipman D. J. Gapped BLAST and PSI-BLAST: a new generation of protein database search programs. Nucleic Acids Res. 1997, 25, 3389–3402. 10.1093/nar/25.17.3389. 9254694 PMC146917

[ref88] Madeira F.; Pearce M.; Tivey A. R. N.; Basutkar P.; Lee J.; Edbali O.; Madhusoodanan N.; Kolesnikov A.; Lopez R. Search and sequence analysis tools services from EMBL-EBI in 2022. Nucleic Acids Res. 2022, 50, W276–W279. 10.1093/nar/gkac240. 35412617 PMC9252731

[ref89] Jacobson M. P.; Friesner R. A.; Xiang Z.; Honig B. On the Role of the Crystal Environment in Determining Protein Side-chain Conformations. J. Mol. Biol. 2002, 320, 597–608. 10.1016/S0022-2836(02)00470-9. 12096912

[ref90] Jacobson M. P.; Pincus D. L.; Rapp C. S.; Day T. J. F.; Honig B.; Shaw D. E.; Friesner R. A. A hierarchical approach to all-atom protein loop prediction. Proteins: Struct., Funct., Bioinf. 2004, 55, 351–367. 10.1002/prot.10613. 15048827

[ref91] Bas D. C.; Rogers D. M.; Jensen J. H. Very fast prediction and rationalization of pKa values for protein-ligand complexes. Proteins: Struct., Funct., Bioinf. 2008, 73, 765–783. 10.1002/prot.22102. 18498103

[ref92] Lu C.; Wu C.; Ghoreishi D.; Chen W.; Wang L.; Damm W.; Ross G. A.; Dahlgren M. K.; Russell E.; Von Bargen C. D.; et al. OPLS4: Improving Force Field Accuracy on Challenging Regimes of Chemical Space. J. Chem. Theory Comput. 2021, 17, 4291–4300. 10.1021/acs.jctc.1c00302. 34096718

[ref93] Bochevarov A. D.; Harder E.; Hughes T. F.; Greenwood J. R.; Braden D. A.; Philipp D. M.; Rinaldo D.; Halls M. D.; Zhang J.; Friesner R. A. Jaguar: A high-performance quantum chemistry software program with strengths in life and materials sciences. Int. J. Quantum Chem. 2013, 113, 2110–2142. 10.1002/qua.24481.

[ref94] Cortis C. M.; Langlois J.-M.; Beachy M. D.; Friesner R. A. Quantum mechanical geometry optimization in solution using a finite element continuum electrostatics method. J. Chem. Phys. 1996, 105, 5472–5484. 10.1063/1.472388.

[ref95] Cortis C. M.; Friesner R. A. An automatic three-dimensional finite element mesh generation system for the Poisson-Boltzmann equation. J. Comput. Chem. 1997, 18, 1570–1590. 10.1002/(sici)1096-987x(199710)18:13<1570::aid-jcc2>3.0.co;2-o.

[ref96] Cortis C. M.; Friesner R. A. Numerical solution of the Poisson-Boltzmann equation using tetrahedral finite-element meshes. J. Comput. Chem. 1997, 18, 1591–1608. 10.1002/(SICI)1096-987X(199710)18:13<1591::AID-JCC3>3.0.CO;2-M.

[ref97] Ringnalda M. N.; Belhadj M.; Friesner R. A. Pseudospectral Hartree-Fock theory: Applications and algorithmic improvements. J. Chem. Phys. 1990, 93, 3397–3407. 10.1063/1.458819.

[ref98] Lu D.; Marten B.; Cao Y.; Ringnalda M. N.; Friesner R. A.; Goddard W. A. Ab initio predictions of large hyperpolarizability push-pull polymers. Julolidinyl-n-isoxazolone and julolidinyl-n-N,N′-diethylthiobarbituric acid. Chem. Phys. Lett. 1995, 242, 543–547. 10.1016/0009-2614(95)00793-4.

[ref99] Lee C.; Yang W.; Parr R. G. Development of the Colle-Salvetti correlation-energy formula into a functional of the electron density. Phys Rev B Condens Matter 1988, 37, 785–789. 10.1103/PhysRevB.37.785. 9944570

[ref100] Becke A. D. Density-functional thermochemistry. III. The role of exact exchange. J. Chem. Phys. 1993, 98, 5648–5652. 10.1063/1.464913.

[ref101] Devlin F. J.; Finley J. W.; Stephens P. J.; Frisch M. J. Ab Initio Calculation of Vibrational Absorption and Circular Dichroism Spectra Using Density Functional Force Fields: A Comparison of Local, Nonlocal, and Hybrid Density Functionals. J. Phys. Chem. 1995, 99, 16883–16902. 10.1021/j100046a014.

[ref102] Grimme S.; Antony J.; Ehrlich S.; Krieg H. A consistent and accurate ab initio parametrization of density functional dispersion correction (DFT-D) for the 94 elements H-Pu. J. Chem. Phys. 2010, 132, 154104 10.1063/1.3382344. 20423165

[ref103] Hariharan P. C.; Pople J. A. The influence of polarization functions on molecular orbital hydrogenation energies. Theoretica chimica acta 1973, 28, 213–222. 10.1007/BF00533485.

[ref104] Hughes T. F.; Friesner R. A. Development of Accurate DFT Methods for Computing Redox Potentials of Transition Metal Complexes: Results for Model Complexes and Application to Cytochrome P450. J. Chem. Theory Comput. 2012, 8, 442–459. 10.1021/ct2006693. 26596595

[ref105] Harding L. B.; Goddard W. A. Generalized valence bond description of the low-lying states of formaldehyde. J. Am. Chem. Soc. 1975, 97, 6293–6299. 10.1021/ja00855a001.

[ref106] Fischer T. H.; Almlof J. General methods for geometry and wave function optimization. J. Phys. Chem. 1992, 96, 9768–9774. 10.1021/j100203a036.

[ref107] Muller R. P.; Langlois J. M.; Ringnalda M. N.; Friesner R. A.; Goddard W. A. A generalized direct inversion in the iterative subspace approach for generalized valence bond wave functions. J. Chem. Phys. 1994, 100, 1226–1235. 10.1063/1.466653.

[ref108] Friesner R. A.; Banks J. L.; Murphy R. B.; Halgren T. A.; Klicic J. J.; Mainz D. T.; Repasky M. P.; Knoll E. H.; Shelley M.; Perry J. K.; et al. Glide: A New Approach for Rapid, Accurate Docking and Scoring. 1. Method and Assessment of Docking Accuracy. J. Med. Chem. 2004, 47, 1739–1749. 10.1021/jm0306430. 15027865

[ref109] Halgren T. A.; Murphy R. B.; Friesner R. A.; Beard H. S.; Frye L. L.; Pollard W. T.; Banks J. L. Glide: A New Approach for Rapid, Accurate Docking and Scoring. 2. Enrichment Factors in Database Screening. J. Med. Chem. 2004, 47, 1750–1759. 10.1021/jm030644s. 15027866

[ref110] Harder E.; Damm W.; Maple J.; Wu C.; Reboul M.; Xiang J. Y.; Wang L.; Lupyan D.; Dahlgren M. K.; Knight J. L.; et al. OPLS3: A Force Field Providing Broad Coverage of Drug-like Small Molecules and Proteins. J. Chem. Theory Comput. 2016, 12, 281–296. 10.1021/acs.jctc.5b00864. 26584231

[ref111] Wang Y.; Zhuang Y.; DiBerto J. F.; Zhou X. E.; Schmitz G. P.; Yuan Q.; Jain M. K.; Liu W.; Melcher K.; Jiang Y.; et al. Structures of the entire human opioid receptor family. Cell 2023, 186, 413–427.e17. 10.1016/j.cell.2022.12.026. 36638794

[ref112] Yao X.-Q.; Grant B. J. Domain-Opening and Dynamic Coupling in the *α*-Subunit of Heterotrimeric G Proteins. Biophys. J. 2013, 105, L08–L10. 10.1016/j.bpj.2013.06.006. 23870276 PMC3714883

[ref113] Grant B. J.; McCammon J. A.; Caves L. S. D.; Cross R. A. Multivariate Analysis of Conserved Sequence-Structure Relationships in Kinesins: Coupling of the Active Site and a Tubulin-binding Sub-domain. J. Mol. Biol. 2007, 368, 1231–1248. 10.1016/j.jmb.2007.02.049. 17399740

[ref114] Shields G. C.; Laughton C. A.; Orozco M. Molecular Dynamics Simulations of the d(T·A·T) Triple Helix. J. Am. Chem. Soc. 1997, 119, 7463–7469. 10.1021/ja970601z.

[ref115] Shields G. C.; Laughton C. A.; Orozco M. Molecular Dynamics Simulation of a PNA·DNA·PNA Triple Helix in Aqueous Solution. J. Am. Chem. Soc. 1998, 120, 5895–5904. 10.1021/ja9723444.

[ref116] Rasmussen S. G. F.; DeVree B. T.; Zou Y.; Kruse A. C.; Chung K. Y.; Kobilka T. S.; Thian F. S.; Chae P. S.; Pardon E.; Calinski D.; et al. Crystal structure of the β2 adrenergic receptor–Gs protein complex. Nature 2011, 477, 549–555. 10.1038/nature10361. 21772288 PMC3184188

[ref117] Hua T.; Li X.; Wu L.; Iliopoulos-Tsoutsouvas C.; Wang Y.; Wu M.; Shen L.; Brust C. A.; Nikas S. P.; Song F.; et al. Activation and Signaling Mechanism Revealed by Cannabinoid Receptor-G_i_ Complex Structures. Cell 2020, 180, 655–665.e18. 10.1016/j.cell.2020.01.008. 32004463 PMC7898353

[ref118] Scheerer P.; Park J. H.; Hildebrand P. W.; Kim Y. J.; Krauß N.; Choe H.-W.; Hofmann K. P.; Ernst O. P. Crystal structure of opsin in its G-protein-interacting conformation. Nature 2008, 455, 497–502. 10.1038/nature07330. 18818650

[ref119] Lebon G.; Warne T.; Edwards P. C.; Bennett K.; Langmead C. J.; Leslie A. G. W.; Tate C. G. Agonist-bound adenosine A2A receptor structures reveal common features of GPCR activation. Nature 2011, 474, 521–525. 10.1038/nature10136. 21593763 PMC3146096

[ref120] Xu P.; Huang S.; Zhang H.; Mao C.; Zhou X. E.; Cheng X.; Simon I. A.; Shen D.-D.; Yen H.-Y.; Robinson C. V.; et al. Structural insights into the lipid and ligand regulation of serotonin receptors. Nature 2021, 592, 469–473. 10.1038/s41586-021-03376-8. 33762731

[ref121] García-Nafría J.; Nehmé R.; Edwards P. C.; Tate C. G. Cryo-EM structure of the serotonin 5-HT1B receptor coupled to heterotrimeric Go. Nature 2018, 558, 620–623. 10.1038/s41586-018-0241-9. 29925951 PMC6027989

[ref122] Yu J.; Kumar A.; Zhang X.; Martin C.; Raia P.; Koehl A.; Laeremans T.; Steyaert J.; Manglik A.; Ballet S.; Structural Basis of μ-Opioid Receptor-Targeting by a Nanobody Antagonist. bioRxiv 2023, 2023.2012.2006.570395. 10.1038/s41467-024-52947-6 PMC11464722 39384768

[ref123] Robertson M. J.; Papasergi-Scott M. M.; He F.; Seven A. B.; Meyerowitz J. G.; Panova O.; Peroto M. C.; Che T.; Skiniotis G. Structure determination of inactive-state GPCRs with a universal nanobody. Nat. Struct. Mol. Biol. 2022, 29, 1188–1195. 10.1038/s41594-022-00859-8. 36396979 PMC12014012

[ref124] Settimo L.; Bellman K.; Knegtel R. M. Comparison of the accuracy of experimental and predicted pKa values of basic and acidic compounds. Pharm. Res. 2014, 31, 1082–1095. 10.1007/s11095-013-1232-z. 24249037

[ref125] Kaufman J. J.; Semo N. M.; Koski W. S. Microelectrometric titration measurement of the pKa’s and partition and drug distribution coefficients of narcotics and narcotic antagonists and their pH and temperature dependence. J. Med. Chem. 1975, 18, 647–655. 10.1021/jm00241a001. 239235

[ref126] Liptak M. D.; Gross K. C.; Seybold P. G.; Feldgus S.; Shields G. C. Absolute pK(a) determinations for substituted phenols. J. Am. Chem. Soc. 2002, 124, 6421–6427. 10.1021/ja012474j. 12033873

[ref127] Scott C. E.; Pickering E. V.; Anderson G. T. Computational Analysis of the Structure of the Kappa-Opioid Receptor for the Development of Selective Antagonists. In Computational Analysis of the Structure of the Kappa-Opioid Receptor for the Development of Selective Antagonists; American Chemical Society, 2022; Vol. 1428, pp 99–122 10.1021/bk-2022-1428.ch007.

[ref128] Shields G. C. The Molecular Education and Research Consortium in Undergraduate Computational Chemistry (MERCURY): Twenty Years of Exceptional Success Supporting Undergraduate Research and Inclusive Excellence. Spur-Scholarship and Practice of Undergraduate Research 2020, 3, 5–15. 10.18833/spur/3/2/1.

[ref129] Shields G. C. Twenty years of exceptional success: The molecular education and research consortium in undergraduate computational chemistry (MERCURY). Int. J. Quantum Chem. 2020, 120, e26274 10.1002/qua.26274.

