# Supplementary material for: Impact of Intracellular Proteins on μ-Opioid Receptor Structure and Ligand Binding
Source: J Phys Chem B. 2024 Dec 19;129(1):71–87. doi: 10.1021/acs.jpcb.4c05214 (PMC11726672; doi:10.1021/acs.jpcb.4c05214)
Supplement: Supplementary file 1 — jp4c05214_si_002.zip [file jp4c05214_si_002.zip › CBS Extrapolation Formulas.docx]

**DERIVATION OF COMPLETE BASIS SET LIMIT EXTRAPOLATION FORMULAS**

We present the derivations of the three-point complete-basis set extrapolations used to compute DLPNO-CCSD(T)/CBS electronic energies using the cc-pVDZ, cc-pVTZ, and cc-pVQZ basis sets.

**INVERSE 4 – 5 POLYNOMIAL SCHEME**

The inverse 4-5 polynomial expansion is based on the formula

$$E_{N}=E_{CBS}+\frac{a}{{(N+1)}^{4}}+\frac{b}{{(N+1)}^{5}}$$

where $N$ is the highest angular momentum number, $E_{N}$ is the energy corresponding to the highest angular momentum basis set, and $a$ and $b$ are parameters to be determined. *N* = 2, 3, and 4 for the cc-pVDZ, cc-pVTZ, and cc-pVQZ basis sets, and give

$$E_{2}=E_{234}+\frac{a}{81}+\frac{b}{243}$$

$$E_{3}=E_{234}+\frac{a}{256}+\frac{b}{1024}$$

$$E_{4}=E_{234}+\frac{a}{625}+\frac{b}{3125}$$

This set of three equations can be written as

$$\left( \begin{matrix} 1 & {81}^{-1} & {243}^{-1} \\ 1 & {256}^{-1} & {1024}^{-1} \\ 1 & {625}^{-1} & {3125}^{-1} \end{matrix} \right)\left( \begin{matrix} E_{234} \\ a \\ b \end{matrix} \right)=\left( \begin{matrix} E_{2} \\ E_{3} \\ E_{4} \end{matrix} \right)$$

Solving the equation gives the CBS extrapolation formula

$$E_{234}=\frac{1}{1320}(243E_{2}-2048E_{3}+3125E_{4})$$
