# Supplementary material for: Impact of Intracellular Proteins on μ-Opioid Receptor Structure and Ligand Binding
Source: J Phys Chem B. 2024 Dec 19;129(1):71–87. doi: 10.1021/acs.jpcb.4c05214 (PMC11726672; doi:10.1021/acs.jpcb.4c05214)
Supplement: Supplementary file 1 — jp4c05214_si_002.zip [file jp4c05214_si_002.zip › Table of Contents.docx]

**Supporting Information**

Impact of Intracellular Proteins on μ-opioid Receptor Structure and Ligand Binding

*Caitlin E. Scott,^1,2^* Leah A. Juechter,^3^ Josephine Rocha,^1^ Lauren D. Jones^3^ Brenna Outten,^3^ Taylor D. Aishman,^2^ Alaina R. Ivers,^2^ George C. Shields^3,^**

^1^Department of Chemistry and Biochemistry, California State University, Los Angeles, Los Angeles, CA, USA.

^2^ Department of Chemistry, Hendrix College, Conway, AR, USA.

^3^Department of Chemistry, Furman University, Greenville, SC, USA.

*Corresponding authors

Section 1: Energetics of Structures

Section 2: Optimized Geometries

Section 3: Derivation of Complete Basis Set Limit Extrapolation Formula

Section 4: Best Glide docking score complexes of small molecule bound to μ-opioid receptors (MOR)

**Section 1: Energetics of Structures**

The electronic energies, G correction values, DLPNO-CCSD(T) electronic energies with the cc-pVnZ basis sets (n=D,T,Q), and complete basis set extrapolations are present in excel files under the folder titled “Energetics of Structures”. The energies are separated into categories based on the ligand and ordered in terms of ascending relative Gibbs free energy.

**Section 2: Optimized Geometries**

The ωB97X-D/6-31++G**/SMD coordinate files of all structures within three kcal∙mol^-1^ of the ∆G˚ minimum for each ligand are presented as .xyz files within the folder titled “XYZ Files”. The .xyz files are organized into different folders based on the ligand and named in order of ascending Gibbs free energy.

**Section 3: Derivation of Complete Basis Set Limit Extrapolation Formulas**

The CBS extrapolation formulas are shown in the word document titled “CBS Extrapolation Formulas”.

**Section 4: Best Glide docking score complexes of small molecule bound to μ-opioid receptors (MOR)**

The coordinate files of the most favorable (lowest) docking scores for each ligand/MOR docking combination are presented as *.mae files in the folder titled “Docking complexes.” The file name format is Fig#_LigandName_MORPDBID.mae.
